# Tumor-targeted IL2 promotes specific CD8^+^ T cells private clonal expansion enhancing lymphoma control

**DOI:** 10.1186/s13046-026-03678-7

**Published:** 2026-03-03

**Authors:** T. Virgilio, K. Chahine, H. Bansal, C. Pizzichetti, L. L. Renner, A. Capucetti, G. Bilato, I. Latino, D. Morone, A. Pulfer, P. Ventura, F. Mele, E. Puca, D. Mangani, C. Junqueira, F. Sallusto, D. Neri, R. De Luca, S. F. Gonzalez

**Affiliations:** 1 Faculty of Biomedical Sciences, Università Della Svizzera italiana, Institute for Research in Biomedicine, Bellinzona, Switzerland; 2 Graduate School for Cellular and Biomedical Sciences, University of Bern, Bern, Switzerland; 3 Department of Biotechnology and Life Sciences, Laboratory of Immunology and General Pathology, University of Insubria, Varese, Italy; 4 Unit of Molecular Pathology, Biochemistry and Immunology, IRCCS MultiMedica, Milan, Italy; 5 Philochem AG, Otelfingen, Switzerland; 6 Instituto René Rachou, Fundação Oswaldo Cruz, Belo Horizonte, Brazil; 7 Department of Biology, Institute of Microbiology, Swiss Federal Institute of Technology (ETH Zürich), Zurich, Switzerland; 8 Philogen S.p.A., Siena, Italy

**Keywords:** Immunocytokine, IL2, CD8^+^ T cells, Immunotherapy, Diffuse large B cell lymphoma, Intravital microscopy

## Abstract

**Background:**

L19IL2 is a clinical-stage antibody-cytokine fusion protein that has been investigated for the treatment of various cancer types. Despite its promising antitumor activity, the precise mechanism of action is still not fully understood.

**Methods:**

In this work, we employed a *myc-*driven B-cell lymphoma murine model to demonstrate that systemic administration of L19IL2 induced a robust CD8⁺ T cell-dependent tumor regression across multiple organs without expansion of regulatory T cells.

**Results:**

Following L19IL2 administration, intratumoral CD8^+^ T cells proliferated and acquired effector and memory phenotypes, associated with private clonal expansion and enhanced killing. Moreover, the spatial behavior of peritumoral CD8^+^ T cells studied by intravital microscopy demonstrated a rapid increase in tumor-directed motility and infiltration following L19IL2 administration.

**Conclusions:**

These findings described the detailed mechanism of action of L19IL2 against B cell lymphoma and revealed for the first time the dynamic responses of peritumoral CD8^+^ T cells to targeted IL2 stimulation, supporting the use of L19IL2 for patients with aggressive B cell lymphoma.

**Supplementary Information:**

The online version contains supplementary material available at 10.1186/s13046-026-03678-7.

## Introduction

B-cell lymphomas represent a diverse group of hematologic malignancies originating from B lymphocytes and are among the most common cancers affecting both the lymphatic and extranodal systems [1]. Their clinical behavior ranges from indolent to highly aggressive subtypes, with diffuse large B-cell lymphoma (DLBCL) being one of the most frequent forms [2]. DLBCL is defined by high aggressiveness and is clinically heterogeneous, with approximately 40% of patients failing to achieve remission.

The standard of care for DLBCL patients is the combination of rituximab, cyclophosphamide, doxorubicin hydrochloride (hydroxydaunorubicin), vincristine sulfate (Oncovin), and prednisone (R-CHOP) [3]. However, while this chemoimmunotherapy has significantly improved the outcome for many patients over the last two decades, therapeutic resistance and relapse remain common challenges [4]. Unlike solid tumors, DLBCL often shows limited or absent expression of programmed death-1 (PD1) or its ligands, reducing the efficacy of immune checkpoint blockade therapies [5, 6]. Although chimeric antigen receptor (CAR) T-cell therapies have shown promising results in relapsed or refractory B-cell lymphomas [7], they are not without limitations, including high cost, limited accessibility, and severe toxicities. Consequently, the development of novel, targeted immune-based strategies remains an urgent clinical need, particularly for patients with relapsed disease or those ineligible for CAR T therapy.

The targeted delivery of bioactive payloads by means of antibodies specific to tumor-associated antigens represents a promising strategy for the development of selective antineoplastic agents [8]. This approach is especially advantageous in lymphomas, for which tumor detection and treatment using conventional methods is often challenging due to their spread dissemination pattern through the organism [9]. In addition to antigens expressed on the tumor cell surface, markers of neoangiogenesis in the extracellular matrix have been considered for targeted pharmacodelivery. Amongst them, the alternatively spliced extra domain B (EDB) of fibronectin has been extensively studied. EDB is highly expressed in the majority of malignancies while being almost absent in healthy adult tissues [10, 11]. Moreover, the 91 amino acid sequence of EDB is fully conserved between mice and humans, facilitating translational activities. This allows the use of the fully human monoclonal L19 antibody (specific to EDB) in both preclinical and clinical settings [12, 13].

Cytokines are potent immunomodulatory proteins that can be used to control anticancer immunity [8]. Among cytokines, Interleukin-2 (IL2) has been investigated for the treatment of different types of tumors due to its capacity to activate cytotoxic CD8^+^ T cells and natural killer (NK) cells [14]. Although recombinant IL2 has been approved for the therapy of metastatic melanoma and renal cell carcinoma, its clinical use remains limited by the severe toxicity and suboptimal pharmacokinetic profiles [15].

To improve the therapeutic index of IL2, an antibody-cytokine fusion protein (also named immunocytokine) based on the L19 antibody (L19IL2) has been generated [16]. L19IL2 has been extensively studied in preclinical models of different types of cancer [17, 18] and in clinical trials [19–21], showing superior therapeutic efficacy than IL2 [16, 22].

While L19IL2 has shown promising antitumoral activity in B cell lymphoma, overcoming unconjugated IL2 [23], its mechanism of action has not yet been clarified. It is currently unclear whether L19IL2 primarily induces cytotoxic cell proliferation, enhances killing, or alters clonal composition. Furthermore, the interplay between the immune cell subsets and the lymphoma architecture, as well as the activation dynamics in response to targeted IL2 delivery, remain to be elucidated.

Recent studies have highlighted the importance of CD8⁺ T cell motility within the tumor microenvironment (TME) as a critical determinant of effective antitumor immunity. Infiltrating CD8⁺ T cells must dynamically scan the TME to locate and eliminate malignant cells, a process that depends on their ability to maintain high motility and directional migration [24]. This mechanism depends on IL2, which plays a key role in enhancing the migratory behavior of CD8⁺ T cells by the upregulation of adhesion molecules and chemokine receptors [25], and by cytoskeleton remodeling [26]. Conversely, tumor-infiltrating T cells often exhibit impaired motility due to chronic antigen exposure and metabolic stress, a limitation that can be reversed by IL2-based immunotherapies [27].

However, several questions are still unanswered, including how CD8^+^ T cells motility correlates with tumor killing in response to selective IL2 stimulation. These questions have direct implications for optimizing spatiotemporal immune responses in malignancies such as lymphoma.

In this study, we developed a murine model of aggressive lymphoma to investigate the therapeutic potential and immunological mechanism of action of L19IL2. We report that targeted delivery of IL2 via the L19 antibody elicits a potent antitumoral response and explore the pathways mediating this effect at both the molecular and motility levels. By elucidating the immunomodulatory landscape shaped by L19IL2 in lymphoma, our findings provide critical insights into the clinical application of this therapy for the treatment of aggressive lymphomas.

## Materials and methods

### Mice

For the majority of experiments we used C57BL/6 J (B6) mice bred in-house or acquired from Charles River Laboratories. CD45.2 HZ mice (B6.SJL-*Ptprc*^*a*^*Pepc*^*b*^/BoyJ, JAX stock 002014) and Ncr1-GFP mice (B6;129-*Ncr1*^*tm1Oman*^/J, JAX stock 022739) were acquired from Charles River in 2021 and later bred in-house. The HZ mutation was maintained in a homozygous background, while the Ncr1-GFP knock-in mutation was maintained in heterozygous state by breeding homozygous animals with wild type B6. All mice were kept under specific pathogen-free conditions in the animal facility of the Institute for Research in Biomedicine, housed in Individually Ventilated Cages with controlled light–dark cycle (12:12), humidity (30–70%), and temperature (20–24 °C). Experiments were performed according to the state guidelines and approved by the local ethical committee ("Dipartimento della Sanità e Socialità, Esperimenti su animali" Canton Ticino), authorization number TI-129/2023. Mice from both genders and age ranging from 6 to 10 weeks, showing good health conditions and no abnormal clinical signs were used in experiments. Mice were assigned to groups through a randomized block design before the first administration of L19IL2. Euthanasia was performed by CO_2_ overdose.

### Cell culture

The Eµ-myc560 cell line was donated by the laboratory of Andreas Strasser (University of Melbourne, Australia), who first described these cells [28]. Cells were expanded for 2–3 passages and aliquots were stored at −150 °C. Aliquots were thawed and used for both in in vivo and in vitro experiments within 5 passages from thawing. Cell were maintained at 37 °C in a humidified thermostat with 5% CO_2_, and periodically tested for Mycoplasma (MycoAlert Mycoplasma Detection kit, Lonza, LT07-418). For culture we used a complete DMEM medium composed of DMEM, high glucose, pyruvate, no glutamine (Gibco, 21969035) supplemented with 10% heat-inactivated FBS (Gibco, 10270–106), 1% Hepes (Gibco, 15630–056), 1% nonessential amino acids (Gibco, 11140–035), 50 units/mL penicillin and 50 mg/mL streptomycin (Gibco, 15070–063), and 50 mmol/L β-mercaptoethanol (Gibco, 31350–010). Eµ-myc560-mCherry cells, referred throughout the text as Eµ-myc, were generated by lentiviral transduction, as we previously described [29]. The lentiviral plasmids pSicoR- Ef1a-mCh (Addgene, 31,847) was purified using a Plasmid Maxi Kit (QIAGEN, 12162). We then transfected HEK293T cells (ATCC, CRL-3216) using pMD2G and psPAX (Addgene, 12260 and 12259) as packaging vectors to generate viral particles. After concentration by centrifugation, the virus was collected and used for Eµ-myc transduction. Cells were incubated with different serial dilutions of the virus for 48 h, and the transduction efficiency was checked by flow cytometry quantification of mCherry signal. The condition presenting the highest percentage of mCherry^+^ cells was then selected and mCherry^hi^ cells were sorted using a BD FACSAria Sorter.

### Lymphoma implantation and monitoring

5 × 10^6^ Eμ-myc cells were resuspended in 50 μL of sterile PBS and injected intravenously (i.v.) with a 1 mL syringe equipped with a 26G needle in the lateral tail vein of awake B6 mice. To facilitate the injection, we induced vasodilation by warming up mice with a red lamp for 2–5 min before injection. Mice were then restrained in a tunnel holder during the injection. After injection, mice were checked at least twice per week and body weight was calculated as an indicator of disease progression. In each experiment, all animals were euthanized at the same time, corresponding to the endpoint of 10% body weight loss and the maximum lymphoma spread. For most experiments, this time corresponded to 18 dpi. For some experiments, such as NK and CD8 depletion, this time was anticipated to 15 and 17 dpi, respectively, due to faster tumor growth. For the Image Stream experiment, mice were sacrificed at 16 dpi as the experimental goal was the visualization of DC-CD8 interactions shortly after the last L19IL2 treatment rather than lymphoma quantification. Lymph nodes (popliteal and inguinal), spleen, blood, and bone marrow were harvested in most of the experiments. The specific timing of of euthanasia and the organs collected in each experiment are specified in the corresponding figures.

### In vivo treatments

**Fig. 1:**
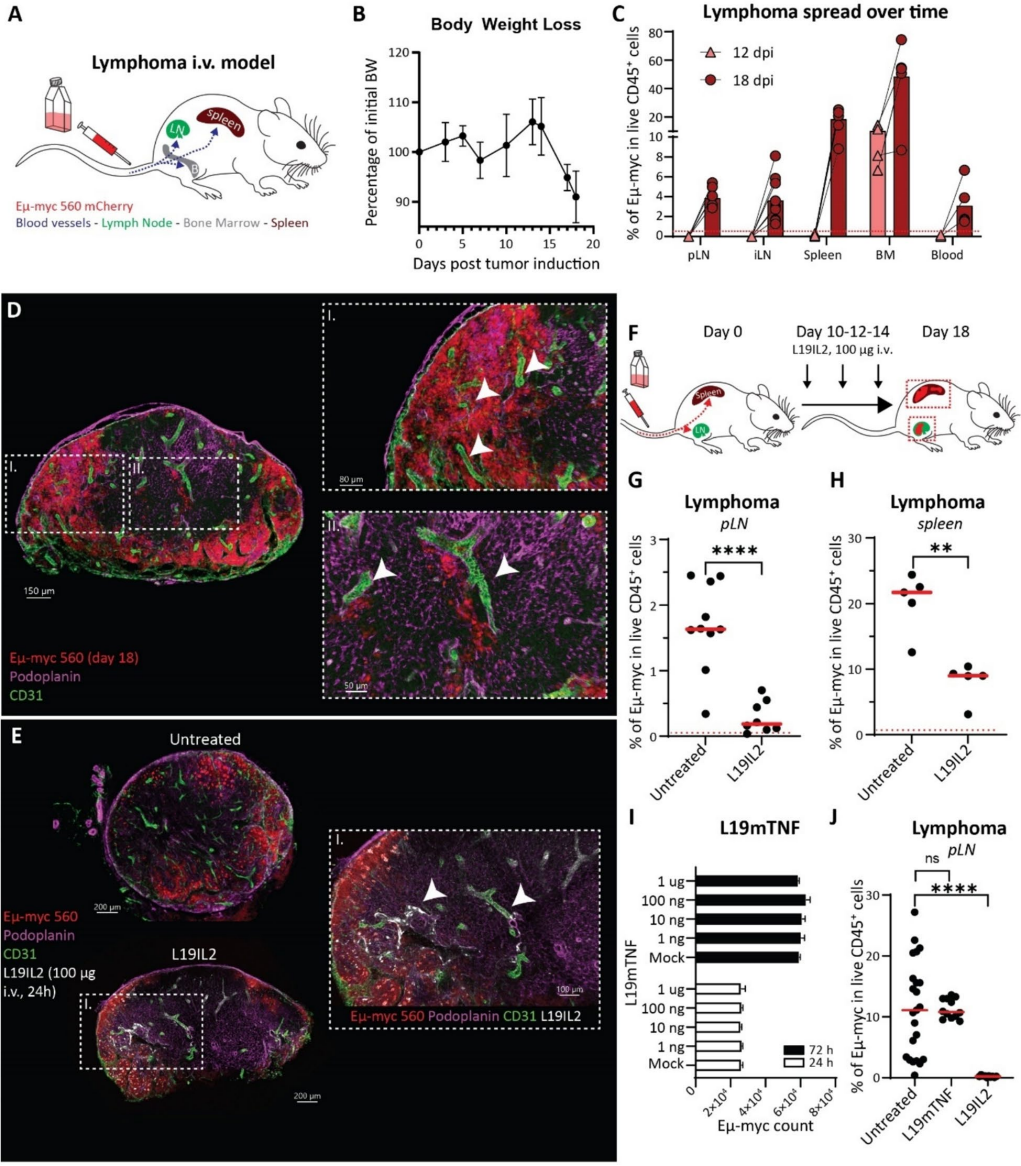
**A** Schematic representation of the lymphoma model, representing the dissemination of lymphoma cells to the different organs after intravenous (i.v.) injection. **B** Body weight (BW) loss, expressed in percentage of initial body weight, in lymphoma bearing mice. **C** Flow cytometric quantification of lymphoma spread into different organs at 12 and 18 days post tumor implantation (dpi). pLN = popliteal lymph node; iLN = inguinal lymph node; BM = bone marrow. **D** Left: representative confocal micrograph showing the invasion of a pLN by lymphoma cells (red) at 18 dpi. Right: magnification of the cortical and paracortical areas (I and II, respectively), showing the infiltration of blood and lymphatic vessels. **E** Confocal micrograph showing the colocalization of L19IL2-AF647 (white) with lymphoma lesions (red) in treated mice compared to untreated controls. The magnifications on the right highlight an area of L19IL2 accumulation. **F** Schematic representation of the L19IL2 administration protocol and organs collected at 18 dpi. Flow cytometric quantifications of lymphoma cells at 18 dpi in L19IL2 treated mice in comparison to untreated mice in the (**G**) pLN and (**H**) spleen (**I**) Flow cytometric count of lymphoma cells co-cultured with different doses of L19mTNF for 24 h (white) or 72 h (black). Mock stands for addition of equivalent volume of cell media without L19mTNF. (*n* = 4). **J **Flow cytometric quantification of lymphoma cells in the pLN of mice treated with L19IL2 or L19mTNF at 18 dpi in comparison to untreated controls. In (**B**) circles indicate average value and line standard deviation. In (**C**), (**G**-**H**), and (**J**) circles represent individual values (animal replicates), lines or bars indicate average per group, and the red dashed lines indicate mCherry background. In all graphs, the *p*-value is indicated as * < 0.05; ** < 0.01; *** < 0.001; **** < 0.0001

L19IL2 and L19mTNF were provided by Philochem AG and described before [30]. For the confocal experiment in Fig. 1E, L19IL2 was conjugated to AlexaFluor647 (ThermoFisher, MP10168) using the Amine-Reactive Probes kit (ThermoFisher, MP00143) and purified with Zeba Dye and Biotin Removal Spin Columns (ThermoFisher, A44298), as per manufacturer’s instructions. L19IL2 was administered i.v. every other day for a maximum of 3 administrations in total volume of 50 µl, diluted in sterile PBS at the dose of 100 µg/mL. For most experiments, the administration dates were 10, 12, and 14 dpi. However, these timepoints were modified in some experiments to coordinate the lymphoma endpoint with either an acute or long-term effect of L19IL2. The specific administration timepoint for each experiment is showed in each corresponding figure. For in vivo depletion of NK cells, mice were treated with 200 µg of anti-NK1.1 (clone BE0036, BioXCell) one day before tumor implantation followed by 100 µg booster doses at 3, 6, and 11 dpi. For in vivo depletion of CD8 T cells, mice were treated with 200 µg of anti-CD8α (clone 53–6.7, BioXCell BE0004-1, RRID AB_1125541) one day before tumor inoculation followed by 150 µg booster doses at 3, 10, and 14 dpi.

### In vitro treatments

To assess the sensitivity of lymphoma cells to TNF, we plated 20*10^3^ Eμ-myc cells per well in a 96 well plate. Six hours later, we added serial dilutions of L19mTNF, indicated in the corresponding figure, in cell media. After 24 or 72 h of incubation, we collected cells and run the entire volume of each well through a FACSymphony (BD Biosciences) to quantify the number of mCherry^+^ cells. To compare the effect of L19IL2 and unconjugated IL2 on the immunophenotype of CD8^+^ T cells, we isolated CD8^+^ T cells from the lymphoid organs of wild type B6 mice using the EasySep Mouse CD8^+^ T cell Isolation Kit (STEMCELL, 19853). Then we stimulated CD8^+^ T cells with αCD3 and αCD28 antibodies in the presence of either IL2 or L19IL2 at equivalent IL2 concentrations (20 International Unit/mL) for three days before collecting them and processing them for flow cytometry as described below.

### Confocal microscopy

For laser scanning confocal microscopy experiments, LNs were fixed in 4% paraformaldehyde (Merck, 818,715) at 4 °C overnight. Then, organs were washed in PBS before embedding in 5% low gelling agarose (Sigma-Aldrich, A9414). Sections (50–70 µm) were cut with Leica VT 1200S vibratome (Leica Microsystems). Slices were stained in a blocking buffer composed of TritonX100 (VWR, 0694) 0.1%, BSA 5% (VWR, 422361 V), and fluorescently labeled antibodies at the appropriate concentration, all diluted in PBS supplemented with calcium and magnesium (DPBS +/+, ThermoFisher, 14040091) shaking at RT overnight. The following day, samples were then washed in 0.05% Tween 20 (Sigma-Aldrich, P7949), when necessary stained 1 h RT with secondary antibodies using the same buffer as for primary antibodies, then washed again in Tween 20, washed in PBS-/-, and mounted on glass slides. Immunofluorescence confocal microscopy was performed using a Leica Stellaris 5 or Stellaris 8 confocal microscope (Leica Microsystems) with a Leica PL APO 10x/NA 0.4 objective. 30 µm z-stacks were acquired with 5 µm step size. Micrographs were acquired in sequential scans and merged to obtain a multicolor image. Images were processed using Imaris software (Bitplane AG).

### Flow cytometry

LNs were collected, disrupted with tweezers, and filtered in 40 µm strainers (ClearLine, 141378 C). Spleens were smashed directly on cell strainers using syringe plungers. Blood and splenocytes suspension were treated with the BD Pharm Lyse Lysing Buffer (BD, 555899), 10 min, shaking at RT, to eliminate Red Blood Cells. BM was collected by flushing one femur and filtering cell suspension. All cells were always handled in a solution of 2 mmol/L EDTA (Sigma-Aldrich, A3145) and 2% heat-inactivated filter-sterilized fetal bovine serum (Gibco, 10270–106) diluted in PBS. We blocked Fc receptors (aCD16/32, BioLegend, 101302), stained dead cells (Zombie Aqua Fixable Viability Kit, BioLegend, 423101), and performed surface staining. Intracellular staining was performed following the surface staining with a dedicated kit (eBioscience, 88/8824/00) according to the manufacturer’s instructions. Stained cells were run through a FACSymphony (BD Biosciences) and data were analyzed using FlowJo 10.10.0 software (FlowJo LLC). For UMAP clustering of T cells, we used the web platform CRUSTY [31]. Briefly, FlowJo gates were downsampled to have a total of 3000 T cells per sample. Files were then exported in the.csv format and imported to CRUSTY. Clusters were determined by applying the PhenoGraph algorithm [31] with a K value of 500, and their expression of each identity marker is reported in the corresponding figure.

### Antibodies

For microscopy and flow cytometry experiments we used the following antibodies: CD45.2-BUV395 (BD, 564279), CD45.1-PerCP-Cy5.5 (BioLegend, 110728), B220-BUV496 (BD, 612950), CD3-PE-Cy7 (BioLegend, 100220), CD4-APC-Cy7 (BioLegend, 100526), CD8a-BV650 (BioLegend, 100742), CD25-PE-Cy7 (eBioscience, 13-0251-85), NK1.1-PerCP-Cy5.5 (Invitrogen, 45-5941-82), Ki67-BV605 (Sirigen, 652413), MHC II-Pacific Blue (BioLegend, 107620), CD11c-BV711 (BioLegend, 101243), CD11b-BV785 (BioLegend, 101243), F4/80-AF488 (BioLegend, 123120), Gr1-APC-Cy7 (BioLegend, 108424), CXCR3-APC (BioLegend, 126511), CD62L-PerCP (BioLegend, 104430), CD44-PE (BioLegend, 103008), CD27-PE-Cy7 (Invitrogen, 25–0271-82), PD1-APC (BioLegend, 108909), Tbet-BV606 (BioLegend, 644817), TNFα-BV785 (BioLegend, 506341), IFNγ-APCFire750 (BioLegend, 505859), TOX-VioB515 (Miltenyi, 130–129–208), TCF1-AF647 (Cell Signaling Technology, 6709S), CD127-PECy7 (ThermoFisher, 25-1273-82), GranzymeB-AF647 (BioLegend, 515405), CD80-PE (BioLegend, 104708), ZAP70(pY319)/Syk(Y352)-PerCP-Cy5.5 (BD, 561459), LCK(pY505)-AF488 (BD, 557879), Akt(pS473)-AF647 (BD, 560343), SHP2(pY542)-PE (BD, 560389), CD169-AF647 (BioLegend, 142408), Podoplanin-PE (Invitrogen, 12-5381-82), and CD31 (Merck, 1398Z). The secondary antibody for CD31 staining was an anti-armenian hamster DyLight 488 (BioLegend, 405503).

### T cell proliferation assay

**Fig. 2:**
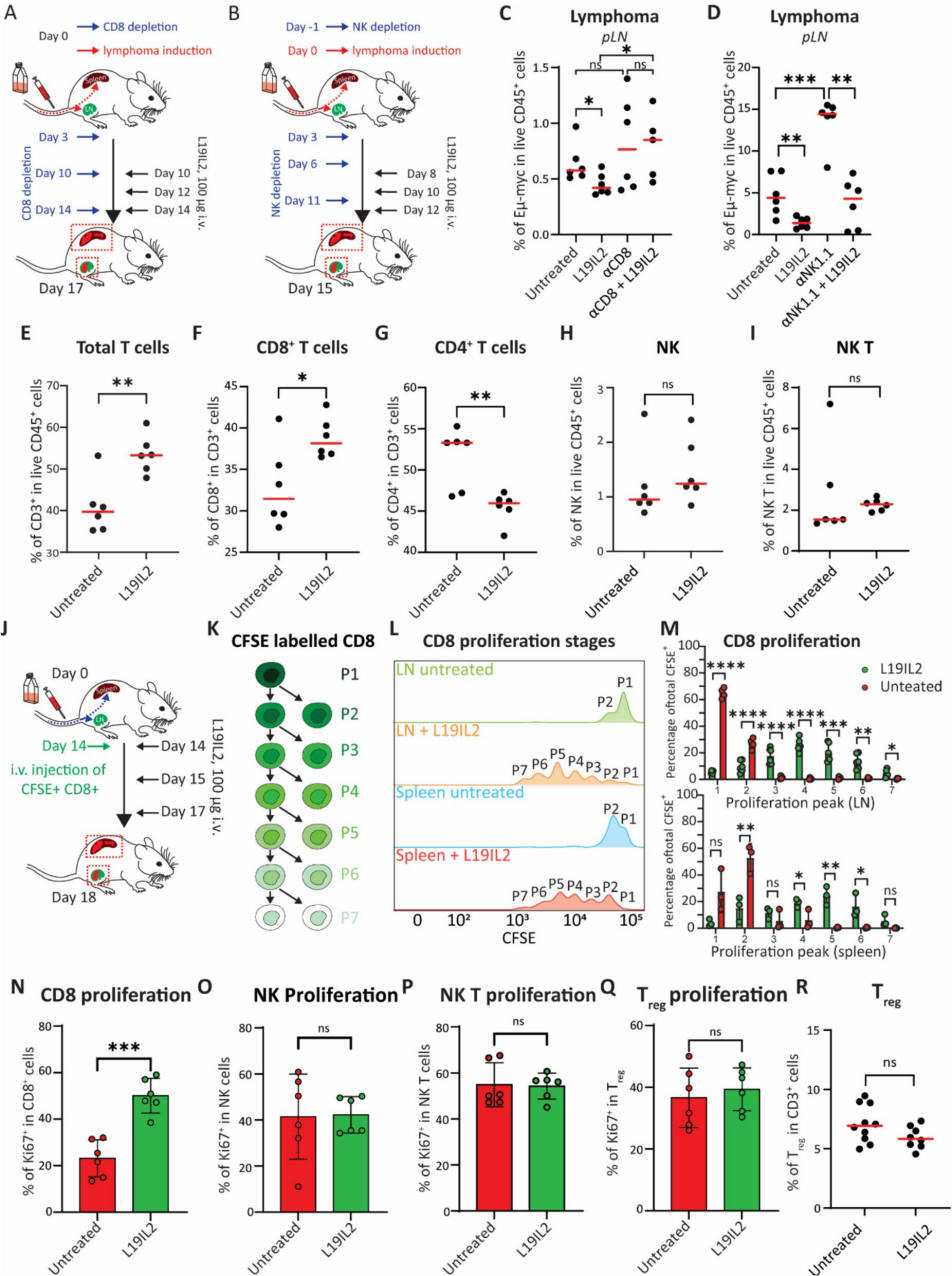
Schematic representation of (**A**) CD8 and (**B**) NK cells depletion models. Flow cytometric quantification of lymphoma cells in the pLN after depletion of (**C**) CD8 or (**D**) NK cells. Flow cytometric quantifications of (**E**) total CD3^+^ T cells, (**F**) CD8^+^ T cells, (**G**) CD4^+^ T cells, (**H**) NK cells, and (**I**) NK T cells in the pLN of untreated and L19IL2 treated mice. **J** Schematic representation of adoptive transfer of CFSE labelled CD8^+^ T cells for in vivo proliferation assay. **K** Schematic representation of the CFSE (green) dilution effect correlated with proliferation events in labelled cells. P1 indicates no proliferation, while P7 corresponds to cells undergoing six proliferation cycles in a sequence. **L** Flow cytometric representative histogram showing the different CFSE peaks in different organs and conditions. **M** Flow cytometry quantification of CD8^+^ T cells proportion for each proliferative peak in the spleen and the pLN of untreated and L19IL2 treated mice (average per group, *n* = 3 in spleen, *n* = 6 in LN). Flow cytometric quantifications of the percentage of Ki67^+^ cells in the LN of untreated and L19IL2 treated mice in (**N**) CD8.^+^ T cells, (**O**) NK cells, (**P**) NK T cells, and (**Q**) regulatory T cells (T_reg_). **R** Flow cytometric quantification of total T_reg_ in the LN of treated and untreated mice. In all the graphs circles represent individual values (animal replicates), while red lines or bars indicate the average value per group. In all graphs, the *p*-value is indicated as * < 0.05; ** < 0.01; *** < 0.001; **** < 0.0001

CD8^+^ T cells were isolated from the spleen of CD45.1 HZ mice using the EasySep Mouse CD8^+^ T cell Isolation Kit (STEMCELL, 19853) and labeled with 5 μM CFSE (ThermoFisher, C34570) for 20 min at 37 °C. Labeled cells were injected i.v. in the tail vein (8.6 * 10^6^ cells per mouse) of L19IL2 or untreated mice 14 dpi, according to the schematic representation in Fig. 2J. The numbers and CFSE intensity of donor cells were measured by flow cytometry as described above.

### RNA extraction and Nanostring analysis

CD8^+^ T cells were isolated from LNs of L19IL2 treated or untreated lymphoma-bearing mice as described above. Then RNA was extracted using the RNeasy Mini Kit (Qiagen, 74106). DNA contaminations were removed with RNase-Free DNase (Qiagen, 79254). mRNA expression was profiled using the nCounter® Immune Exhaustion Panel (NanoString), as per the manufacturer’s instructions, and according to the “Hybridization Protocol for nCounter XT CodeSet Gene Expression Assays Including Panels,” starting from 30 ng of total RNA per sample. Raw values were processed using the nSolver Analysis Software 4.0 (Nanostring). The background thresholds were calculated as the mean of the negative controls plus two times their standard deviation. Samples were normalized on the geometric mean of technical positive controls and biological references (housekeeping). Raw data were log transformed (base 2) for analysis and p value set to < 0.05. Fold change (log) and adjusted p value (-log10 p value) were used for final data representation. For gene sets analysis a list of all the differentially regulated genes was used as an input in g:profiler [32] and the most significant correlated pathways from the Gene Ontology library [33] were represented.

### CD8 + T cells killing assay

CD8^+^ T cells were isolated from LNs of L19IL2 treated or untreated animals as done for RNA extraction. Then, CD8^+^ T cells were co-cultured with 20*10^3^ Eμ-myc cells in different effector:target ratios, including 1:1, 5:1, and 10:1, for 24 or 48 h. Then, lymphoma cells were quantified by flow cytometry as described above.

### TCR sequencing

CD8^+^ T cells were isolated from L19IL2 treated and untreated lymphoma-bearing mice as described above. Next, genomic DNA was isolated using the Micro Dna Isolation Kit (Qiagen, 56304) and shipped to Adaptive Biotechnologies for TCRB immunosequencing after quantification and purity check through spectrophotometric analysis. Sequencing of TCR Vβ CDR3 was performed by Adaptive Biotechnologies using the ImmunoSEQ assay (http://www.immunoseq.com). In brief, following multiplex PCR reaction designed to target any CDR3 Vβ fragments, amplicons were sequenced using the Illumina HiSeq platform. Raw data consisting of all retrieved sequences of 87 nucleotides or corresponding amino acid sequences and containing the CDR3 region were exported and further processed. Each TCR Vβ clonotype was defined as the unique combination of a productively rearranged CDR3 amino acid sequence and its related V and J genes (bioidentity). Data processing was done using the productive frequency of templates provided by ImmunoSEQ Analyzer V.3.0 (http://www.immunoseq.com).

### Image stream

CD8^+^ T cells and DCs were isolated from L19IL2 treated and untreated lymphoma bearing animals as described in Fig. 5F. CD8^+^ T cells were isolated from LNs as described above. DCs were isolated from spleens using a mouse Pan Dendritic Cell Isolation Kit (Miltenyi, 130–100–875). Next, CD8^+^ T cells and DCs were co-cultured in complete RPMI (described above) at a 10:1 CD8:DC ratio, combining L19IL2 treated CD8^+^ T cells with L19IL2 treated DCs or untreated CD8^+^ T cells with untreated DCs. Following 1 h incubation, cells were centrifuged 1500 rpm for 5 min and fixed with 1% PFA for 10 min at room temperature. After three washes in PBS, cells were incubated with an Fc receptor blocker (aCD16/32, BioLegend, 101302) and the CD8-PE-Cy7 (Invitrogen, 25–008–82) and CD11-AF647 (BioLegend, 117312) antibodies for 20 min at 4 °C. Stained doublets were acquired on an ImageStreamX Mark II cytometer (Cytek) and the analysis were performed using the IDEAS 6.4 software (Cytek).

### 2-photon intravital microscopy

For 2-photon intravital microscopy (2P-IVM) of the lymphoma invaded LNs, we injected i.v. CD8^+^ T cells (7*10^6^ cells per mouse) isolated from Naïve B6 mice and labelled with Cell Trace Violet (ThermoFisher, C34557) in recipient lymphoma-bearing Ncr1-GFP mice 12 h before imaging. To visualize the LN structure, a CD169-AF647 (BioLegend, 142408) antibody was injected subcutaneously in the footpad diluted 1/10 in 10 μL PBS 2 h before imaging. Next, the pLN was surgically exposed as previously described [34]. Briefly, mice were anesthetized with isoflurane 30 min after injection of 0.05 mg/kg buprenorphine and 5 min after local subcutaneous injection of lidocaine. Once reached surgical tolerance, animals were positioned on a customized surgical board. After fixing the tail and the leg, we dissected the skin and the subcutaneous fat in the popliteal *fossa,* taking care to avoid damage to blood or lymphatic vessels. Then, we submerged the exposed pLN in PBS and covered it with a glass coverslip for image acquisition. Following a first recording of untreated lymphoma lesions, image acquisition was stopped, L19IL2 (100 μg) was injected i.v. taking care of not moving animals from the image acquisition position. Next, 2P-IVM was re-started to image the same field of view following therapy. At the end of the imaging procedure, animals were euthanized by anesthetic overdose and death confirmation. Deep tissue imaging was performed on a customized upright two-photon platform (TrimScope, LaVision BioTec). Two photon probe excitation and tissue second-harmonic generation (SHG) were obtained with a set of two tunable Ti:sapphire lasers (Chamaleon Ultra II, Coherent) with output wavelength in the range of 690–1080 nm and an optical parametric oscillator that emits in the range of 1,010–1,340 nm (Chamaleon Compact OPO, Coherent). Imaging was performed with a Nikon LWD 16X/0.80 objective. 200 µm z-stacks were acquired with 10 µm step size. Image Analysis and Data Processing Cell detection, tracking and volumetric reconstruction from 4D 2P-IVM data were performed using Imaris (Oxford Instruments, v10.2.0). Cell tracks were generated semi-automatically and curated to correct errors (i.e., jumps or non-detected cells). Tracks with a duration < 5 timeframes (equivalent to 300 s) were excluded from the analysis. Videos were stabilized using translational-drift correction. Standard measures of cell motility were computed using Imaris. These include: Track duration (time interval between the first and the last time points in which a cell is tracked), Track Length (total length of the cell trajectory), Track Speed Mean (Track length/Track duration), Track Displacement (length of the vector from the first to the latest centroid position of the cell), and Track Straightness (Track Displacement/Track Length). The average velocity of pixels was estimated by applying an optical flow [35] based approach to the CD8^+^ T cells channel. The final values are normalized using a Mat2Gray function. Tumor region analysis was performed with ImageJ, by progressively eroding the tumor ROI to create ROIs in shape of multiple inner equidistant ROIs. To identify cells action, we first generate tracklets with 8-point lengths by moving a window across the original full length tracks. This was done using a custom Matlab script which calculated multiparametric cell features including mean speed, mean displacement, mean directionality and mean arrest coefficient. We then used dynamic time-warping alignment technique to evaluate track similarity, as previously described [36]. This algorithm generated a cross-distance matrix by analyzing multivariate time series information with multidimensional feature count table that were reduced to two dimensions with the UMAP for visualization and clustering purposes. To cluster the tracks, we used the k-means clustering algorithm. The algorithm developed for image analysis is deposited on GitHub at https://github.com/Infection-and-Immunity-Group/Cell-Action-Recognition.

### Chemokines quantification

To measure chemokines expression, we first collected L19-IL2 treated and untreated LNs and carefully disrupted them in 75 μL ice-cold PBS. The suspension was centrifuged at 1500 RPM for 5 min and the supernatant was collected. 25 µL supernatant was used for chemokines detection by a LEGENDPlex assay (Mouse Proinflammatory Chemokine Panel and Mouse Inflammation Panel, BioLegend, 740150). Samples were analyzed by flow cytometry on FACSymphony (BD Biosciences) and data were analyzed using the LEGENDplex Data Analysis Software Suite (BioLegend).

### Quantification and statistical analysis

All data are expressed as the mean ± SD or mean including individual values, which correspond to either technical or animal replicates, as indicated in the figure legends. Each graph shows the results of a single experiment and data from independent experiments were not pooled unless specified in the corresponding figure legend. The specific graphical representations are indicated in each individual figure. Data were analyzed using the Mann–Whitney U test or parametric t-test for comparisons between two groups, and by ANOVA (one-way for a single variable, two-way for multiple variables) or Kruskal–Wallis test for comparisons involving multiple groups. Prior to analysis, data distribution was assessed using the Shapiro–Wilk normality test. Significance was calculated using GraphPad Prism 10.4.2 (GraphPad Software). In all graphs, the *p-*value is indicated as * < 0.05; ** < 0.01; *** < 0.001; **** < 0.0001.

### Declaration of generative AI and AI-assisted technologies in the writing process

During the preparation of this work the authors used ChatGPT (GPT-4.5, o4 model, OpenAI, June 2025) and Grammarly (Grammarly Inc., 2025) to assist with grammar and language check. After using these tools, the authors reviewed and edited the content as needed and take full responsibility for the content of the publication.

## Results

### L19IL2 reduces tumor burden in a systemic lymphoma model

To investigate the antitumoral effects of L19IL2, we used a B-cell lymphoma model based on a Eμ-myc-mutated cell line [28] transduced to express the fluorescent reporter mCherry (Supp. Figure 1 A). Eμ-myc mCherry cells were injected intravenously into the tail vein of mice to enable homing and colonization of lymphoid organs (Fig. 1A). Mice started to lose body weight after 15 days post tumor implantation (dpi), reaching a humane endpoint (10% body weight loss) by 18 dpi (Fig. 1B). To monitor disease progression we collected multiple tissues at different time points and we quantified the frequency of mCherry^+^ lymphoma cells by flow cytometry (Supp. Figure 1B). This analysis revealed that lymphoma cells initially colonized the bone marrow (BM) by 12 dpi consequently invading secondary lymphoid organs, including lymph nodes (LNs), spleen, and systemic circulation by 18 dpi (Fig. 1C).

To validate the model for L19 targeting, we performed confocal microscopy of LN sections at 18 dpi confirming the association of lymphoma lesions with high endothelial venules (CD31^+^ podoplanin^–^) and lymphatic vasculature (CD31^+^ podoplanin^+^) (Fig. 1D). Next we conjugated the L19IL2 with AlexaFluor647 (AF647) and evaluated its specificity by intravenous (i.v.) administration to lymphoma-bearing mice at 18 dpi. Confocal imaging showed that L19IL2-AF647 colocalized specifically with the LN regions infiltrated by lymphoma cells and neovessels (Fig. 1E).

We then tested whether L19IL2 exerted an antitumoral effect in lymphoma. Based on established protocols [37], mice received i.v. injections of 100 μg L19IL2 every other day from 10 to 14 dpi (three doses total; Fig. 1F). A flow cytometric quantification of mCherry^+^ cells at 18 dpi revealed a significant reduction in lymphoma growth in treated mice compared with the control group in different organs, including popliteal LN (pLN, Fig. 1G), spleen (Fig. 1H), and BM (Supp. Figure 1 C).

To determine whether the observed antitumoral effect was mediated by IL2 or by the L19 Fv, we identified murine Tumor Necrosis Factor (mTNF) as a control molecule, unable to induce any effect on the growth of Eμ-myc cells in vitro (F ig. 1I). We then treated lymphoma-bearing mice with L19 conjugated to mTNF (L19mTNF) following the same injection protocol used for L19IL2. Flow cytometry analysis showed no significant reduction in lymphoma burden in pLNs (Fig. 1J), spleen (Supp. Figure 1D), or BM (Supp. Figure 1E) following L19mTNF treatment in comparison to untreated controls. These results confirm that the antitumoral effect observed with L19IL2 is mediated by IL2.

### L19IL2 promotes selective CD8⁺ T cell expansion essential for antitumor immunity

To assess the contribution of immune effector populations to the antitumoral activity of L19IL2, we selectively depleted CD8⁺ T cells (Fig. 2A) or NK cells (Fig. 2B) in lymphoma bearing mice and quantified tumor burden following treatment. First, we confirmed an efficient depletion of CD8⁺ (Supp. Figure 2 A) and NK (Supp. Figure 2B) cells in the LN and spleen by flow cytometry. Next, we observed that the therapeutic benefit of L19IL2 was not observed in the context of CD8^+^ T cell depletion, indicating that these cells are essential mediators of the antitumoral response (Fig. 2C). This result was consistently observed in multiple experiments (Supp. Figure 2 C). Conversely, NK cell depletion did not impair tumor control by L19IL2 in neither LN (Fig. 2D) or spleen (Supp. Figure 2D), suggesting a secondary role for NK cells in this model.

To characterize the effect of L19IL2 on the lymphoma infiltrating immune cells, we profiled by flow cytometry the lymphoid tissues from treated and control animals (Supp. Figure 2E). We observed that L19IL2 administration led to an overall expansion of CD3⁺ T cells in the LN (Fig. 2E), driven primarily by increased CD8⁺ T cells (Fig. 2F), with a concomitant reduction in the proportion of CD4⁺ T cells (Fig. 2G). These results further support the primary role of CD8 T cells in our model. Moreover, we detected no significant changes in the abundance of other immune populations, including NK (Fig. 2H) and NKT (F ig. 2I) cells, B cells (Supp. Figure 2 F), dendritic cells (DCs; Supp. Figure 2G), macrophages (Supp. Figure 2H), and neutrophils (Supp. Figure 2I) at the same time point.

To determine whether the expansion of CD8⁺ T cells reflected a proliferative activity, we transferred CFSE labeled CD8⁺ T cells into lymphoma bearing recipients animals either treated or untreated (Fig. 2J). Flow cytometric analysis revealed enhanced proliferation of CD8⁺ T cells in treated animals, as indicated by the presence of multiple CFSE dilution peaks (Fig. 2K). By comparing the number of cells detected in each peak among conditions (Fig. 2L), we observed that L19IL2 promoted a homogeneous proliferation, with up to six cell divisions in both the LN and spleen, whereas CD8⁺ T cells in control mice underwent only one-three divisions (Fig. 2M). Consistently, a flow cytometric quantification of Ki67⁺ cells (Supp. Figure 2 J) revealed a significant increase in proliferating CD8⁺ T cells in L19IL2 treated animals in comparison to untreated controls (Fig. 2N), while no changes were observed in NK (Fig. 2O), NKT (Fig. 2P), or regulatory T cells (T_reg_; Fig. 2Q). Additionally, Total T_reg_ numbers remained unaffected by the treatment (Fig. 2R).

### L19IL2 activates CD8 T cells more potently than unconjugated IL2

**Fig. 3:**
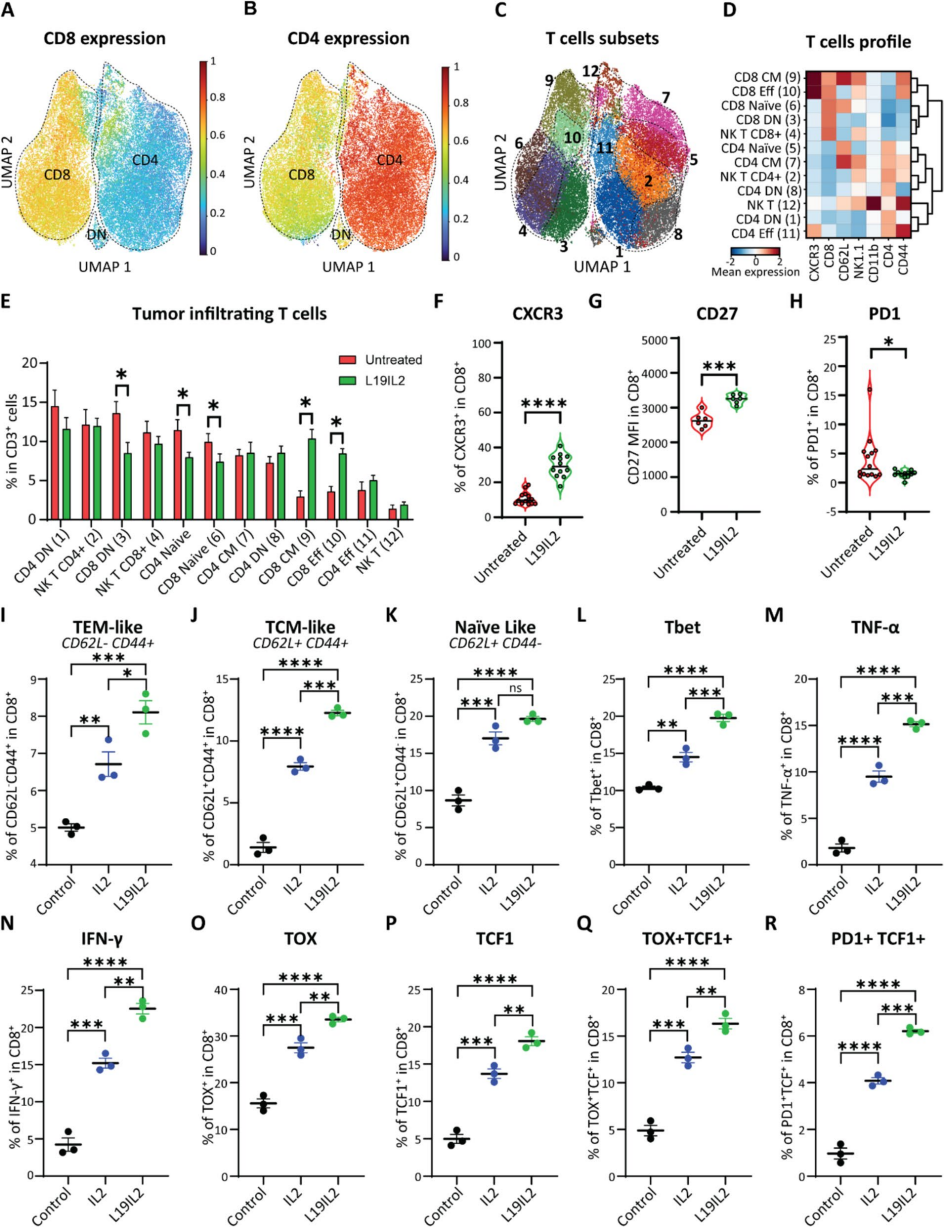
UMAP clustering of total CD3+ T cells acquired by flowcytometry, showing the expression of (**A**) CD8, (**B**) CD4, and (**C**) the subclusters in all animals (6per group, pooled). **D** Heatmap showing average fluorescence expression among the varioussubclusters of the surface expression markers used to generate the UMAP. Numbers on the yaxis indicate the cluster ID, aligned with the cell identity. CM = Central Memory; Eff = Effector; DN= Double Negative for CD44 and CD62L. (**E**) Quantification of the proportion of T cells from eachsubcluster, divided by untreated (red) and L19IL2 treated (green) conditions. Bars indicate theaverage value and lines indicate standard deviation, with 6 animal replicates per group. Violinplots of independent experiments showing the flow cytometric quantification of (**F**) thepercentage of CXCR3+ cells, (**G**) mean CD27 expression, and (**H**) percentage of PD1+ cells amongCD8+ T cells in the LN of untreated and treated animals. Dots indicate values from individualanimals. Percentage in the CD8+ T cells treated in vitro with L19IL2 or IL2 of (**I**) T EffectorMemory like (TEM), (**J**) T Central Memory like (TCM), (**K**) Naϊϊve like, (**L**) Tbet+, (**M**) TNF-α+, (**N**) interferon-γ+ (IFN-γ), **(O)** TOX+, **(P)** TCF1+, **(Q)** double TOX+ TCF1+, and (**R**) double PD1+ TCF1+. In (**A-C**) circles indicate individual cells and all the samples were pooled in the same graph. In (**F**-**R**) circles indicate technical replicates, and lines indicate average and standard deviation pergroup. Control stands for no cytokine treatment. In all graphs, the *p*-value is indicated as * <0.05;** <0.01; ***<0.001; **** < 0.0001

To investigate whether the CD8⁺ T cell expansion observed following L19IL2 therapy was associated with a phenotypic polarization, we performed flow cytometric analysis of LN T cells from treated and control mice (Supp. Figure 3 A). A UMAP analysis of all T cells based on surface marker expression (Supp. Figure 3B) enabled us to distinguish CD8⁺ (Fig. 3A) and CD4⁺ T cells (Fig. 3B), and further separate them into phenotypic subclusters (Fig. 3C). Based on differential marker expression, we defined the following subsets: central memory CD8^+^ (CD62L^+^CD44^+^), effector CD8^+^ (CD62L^–^CD44^+^), naïve CD8^+^ (CD62L^+^CD44^–^), CD8^+^ double negative (CD62L^–^CD44^–^), central memory CD4^+^, effector CD4^+^, naïve CD4^+^, CD4^+^ double negative, NK T CD8^+^ (NK1.1^+^CD11b^–^CD8^+^CD4^–^), NK T CD4^+^, and NK T cells (Fig. 3D). By comparing the composition of the T cell subtypes in the LN of L19IL2 treated (Supp. Figure 3 C) versus untreated control animals (Supp. Figure 3D), we confirmed that the L19IL2 administration significantly increased the proportion of effector and memory CD8⁺ T cells, while reducing double negative CD8, naïve CD8⁺, and naïve CD4⁺ (Fig. 3E). Furthermore, CD8^+^ T cells in L19IL2 treated mice exhibited increased CXCR3 expression (Fig. 3F), higher CD27 levels (Fig. 3G), and decreased PD1 expression, despite its low basal level (Fig. 3H).

Next, to compare direct effects of L19IL2 and unconjugated IL2, we cultured activated (αCD3 and αCD28) CD8⁺ T cells from tumor free mice with either L19IL2 or IL2 at equivalent IL2 concentrations (20 U/mL) for 3 days (Supp. Figure 3E). A phenotypic characterization by flow cytometry revealed that L19IL2 treated cells displayed a higher proportion of effector memory–like (F ig. 3I) and central memory–like (Fig. 3J) populations compared to IL2 treated, but not naïve-like cells (Fig. 3K). Moreover, L19IL2 induced greater expression of effector-associated molecules including T-bet (Fig. 3L), TNF-α (Fig. 3M), IFN-γ (Fig. 3N), and TOX (Fig. 3O) than unconjugated IL2. Expression of CD127 was increased in the treated group compared to the controls but not to the IL2 treated cells (Supp. Figure 3 F). Finally, L19IL2 enhanced the frequency of stem-like (TCF1⁺; Fig. 3P), early activated (TCF1⁺TOX⁺; Fig. 3Q), and late differentiated (TCF1⁺PD1⁺; Fig. 3R) CD8⁺ subsets compared to both IL2 and controls.

### L19IL2 enhances CD8⁺ T cell cytotoxic programs

**Fig. 4:**
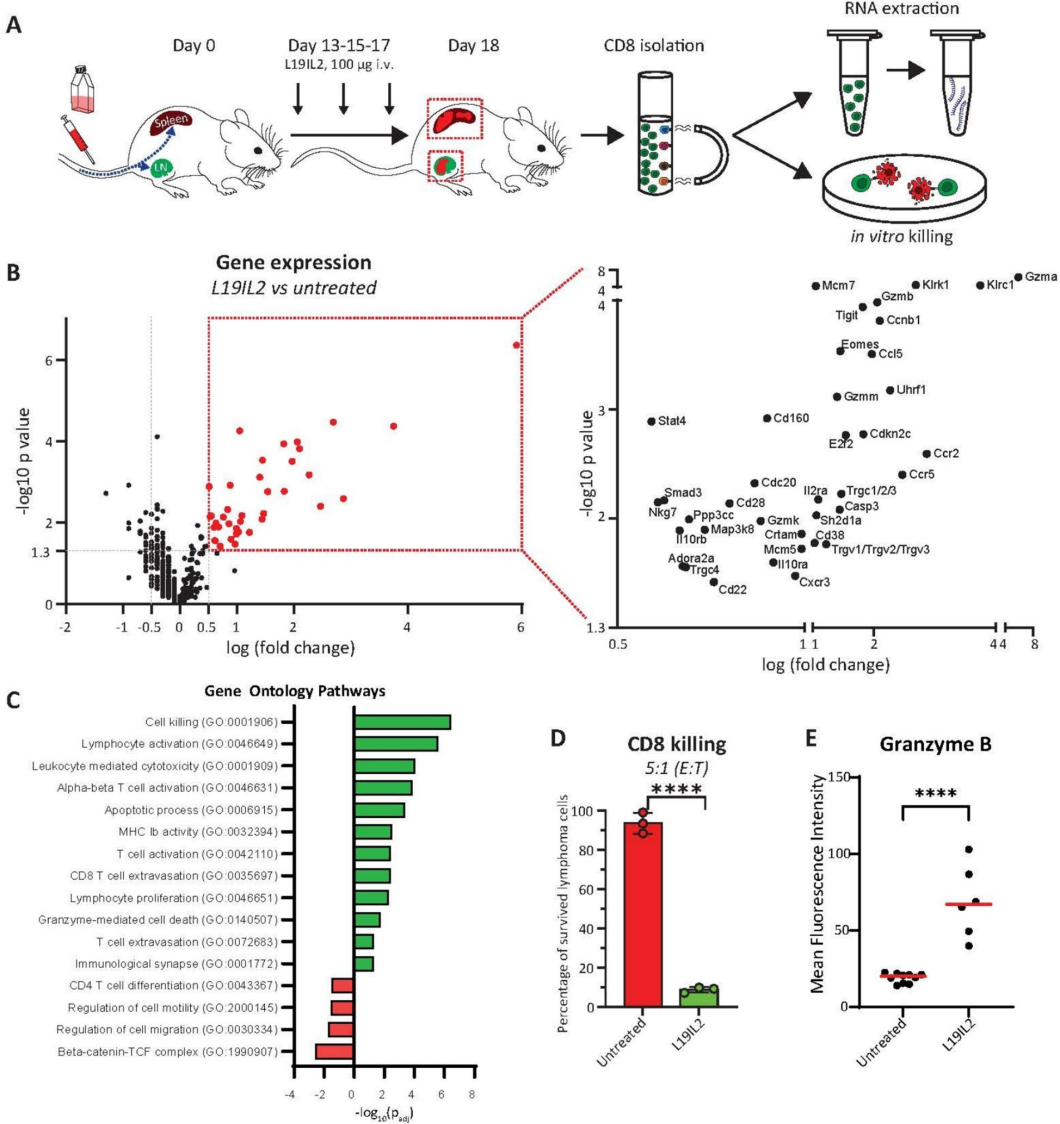
**A** Schematic representation of the experimental pipeline to isolate CD8^+^ T cells for RNA sequencing and in vitro killing experiments. **B** Volcano plot representing the differential gene expression in CD8^+^ T cells isolated from L19IL2 treated mice in comparison to untreated. The dashed vertical lines represent the fold change threshold of 0.5 (genes upregulated in L19IL2) or −0.5 (genes downregulated in L19IL2). The horizontal dashed line represents the statistically significant threshold. The red dashed box contains all the significantly upregulated genes. **C** Bar plot showing the gene ontology pathways with higher statistical association with the upregulated (green) or downregulated (red) genes after IL19IL2 treatment. **B** and **C** show results from 4 animals per group. **D** CD8^+^ T cell killing capacity, measured as the percentage of surviving lymphoma cells, quantified by flow cytometry, following 24 h co-incubation. E:T stands for the ratio in the numbers of plated effector(CD8):target(lymphoma) cells. **E** Flow cytometric quantification of the mean expression of granzyme B in CD8.^+^ T cells isolated from untreated or L19IL2 mice. In (**D**) dots represent technical replicates and bars represent average value per group. In (**E**) dots indicate animal replicates and lines show the average value per group. In all graphs, the *p*-value is indicated as * < 0.05; ** < 0.01; *** < 0.001; **** < 0.0001

To assess whether L19IL2 enhances CD8⁺ T cells cytotoxic machinery, we isolated CD8⁺ T cells from LN of treated and control mice to profile RNA expression and test CD8⁺ T cells killing capacity in vitro (Fig. 4A). By analysing the transcriptomic profile of CD8⁺ T cells based on a pre-selected panel of genes related to T cells functionality (Nanostring, nCounter® Immune Exhaustion Panel) we observed 38 upregulated genes (> 0.5-fold) in L19IL2-treated cells, including genes associated with activation (*Cd160*, *Cd28*, *Cxcr3*, *Eomes*), cytotoxicity (*Gzma*, *Gzmb*), and IL2 signaling (*Il2ra*, *Map3k8*, *Stat4*) (Fig. 4B). We next confirmed by a gene ontology analysis (gProfiler) the enrichment in pathways related to T cell killing, activation, cytotoxicity, MHC and TCR engagement, proliferation, granzyme mediated cell death and apoptosis, T cell extravasation, and immunological synapse (Fig. 4C). In contrast, downregulated genes were enriched in pathways involved in CD4⁺ differentiation and negative regulation of migration (Sup. Figure 4A).

Functionally, CD8⁺ T cells from L19IL2-treated animals exhibited enhanced cytotoxicity, reducing lymphoma cell viability by > 90% after 24 h co-culture compared to controls (Fig. 4D). A similar observation was recorded after 48 h of co-incubation (Supp. Figure 4B) and 24 h and 48 h using CD8⁺ T cells isolated from murine spleen (Supp. Figure 4 C). Further supporting their increased killing capacity, CD8⁺ T cells in L19IL2 treated mice overexpressed granzyme B at a protein level, as indicated by a flow cytometry quantification (Fig. 4E).

### L19IL2 promotes CD8⁺ private clonal expansion via enhanced priming

**Fig. 5:**
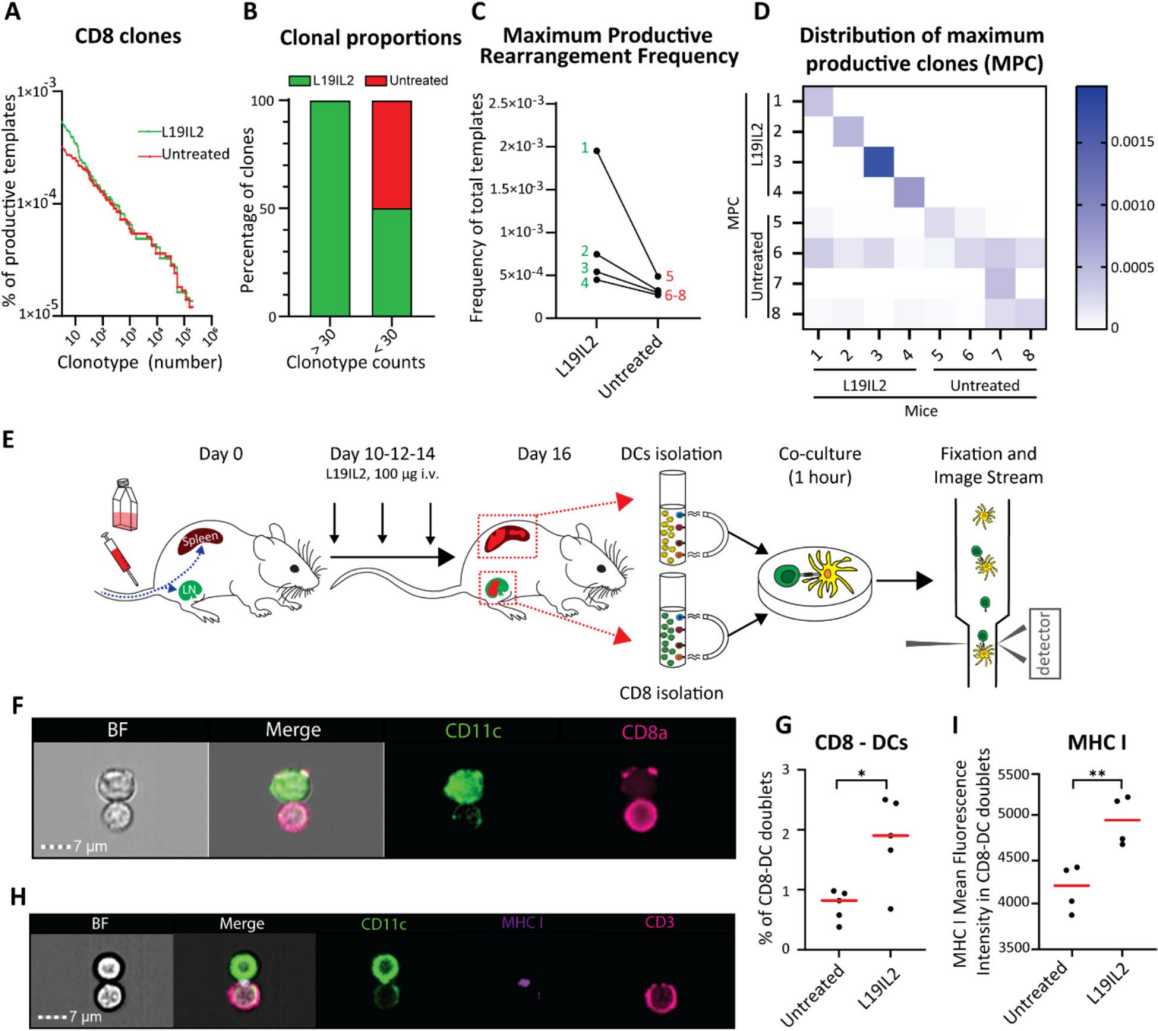
**A** Waterfall graph representing the frequency of all the productive templates in the L19IL2 and untreated groups, ranked from higher (left) to lower (right) frequency. **B** Barplot showing the composition of the more expanded (> 30 clonotype counts) and less expanded (< 30 clonotype counts) clones. Green and red show the percentage of clones belonging to the L19IL2 or untreated group, respectively. **A** and **B** show pooled data from 4 animals for each group. **C** Frequency of the maximum productive clone (i.e. the clone with the highest clonotype counts; MPC) identified in each animal from the L19IL2 (green) and untreated (red) groups. **D** Heatmap showing the frequency of each animal MPC (y axis) across all the other mice (x axis). **E** Schematic representation of the experimental pipeline to quantify CD8-DC doublets by Image Stream. **F** Representative imaging flow cytometry snapshot showing a CD8-DC doublet. **G** Quantification of the percentage of CD8-DC doublets measured in L19IL2 versus untreated condition. **H** Representative imaging flow cytometry snapshot showing MHC I engaging. **I** Quantification of the Mean Fluorescence Intensity of MHC I in DC-CD8.^+^ T cells doublets. In (**G**) and (**I**) dots represent individual values (animal replicates) and red lines indicate the average value per group. In all graphs, the *p*-value is indicated as * < 0.05; ** < 0.01; *** < 0.001; **** < 0.0001

To determine whether L19IL2 induces CD8⁺ T cell clonal expansion, we performed TCR sequencing on CD8⁺ T cells isolated from LN of treated and control mice (Supp. Figure 5 A). Following this approach, we quantified the percentage of each CD8 clone in L19IL2 treated mice versus untreated controls (Fig. 5A) and observed that all highly proliferative clones (> 30 counts) were derived from L19IL2 treated mice, whereas low proliferative clones (< 30 counts) were equally distributed among the groups (Fig. 5B). Moreover, for each animal belonging to the treated and untreated groups, we identified the maximum productive clone (MPC), which corresponds to the most proliferative clone per animal (Supp. Figure 5B). By measuring their counts we observed that the MPC frequency was higher in the L19IL2 group in comparison to untreated controls (Fig. 5C). Next, we analyzed the expression of each MPC across all the animals from both groups (Fig. 5D). This comparison showed that MPC from L19IL2 treated mice were expanded only in the corresponding individual animal, consistently with a private clonal expansion. Conversely, 75% of the untreated MPC (3 out of 4 animals) corresponded to public clones, detected also in all the other animals regardless of the treatment regimen.

To investigate whether this expansion was due to increased antigen priming and the formation of CD8–DC doublets, we co-cultured CD8⁺ T cells with DCs isolated from treated or untreated mice (Fig. 5E). Using imaging flow cytometry (Fig. 5F), we observed that L19IL2 significantly increased doublet formation (Fig. 5G), suggesting enhanced CD8⁺–DC interactions. To confirm enhanced priming, we confirmed that these interactions occurred through MHC I (Fig. 5H), which was upregulated by L19IL2 (F ig. 5I). Consistently, CD28 (Supp. Figure 5 C) and its ligand CD80 (Supp. Figure 5D) were upregulated in CD8⁺ T cells and DCs, respectively, following L19IL2.

### L19IL2 facilitates dynamic CD8⁺ T cells infiltration in the core of lymphoma lesions

**Fig. 6:**
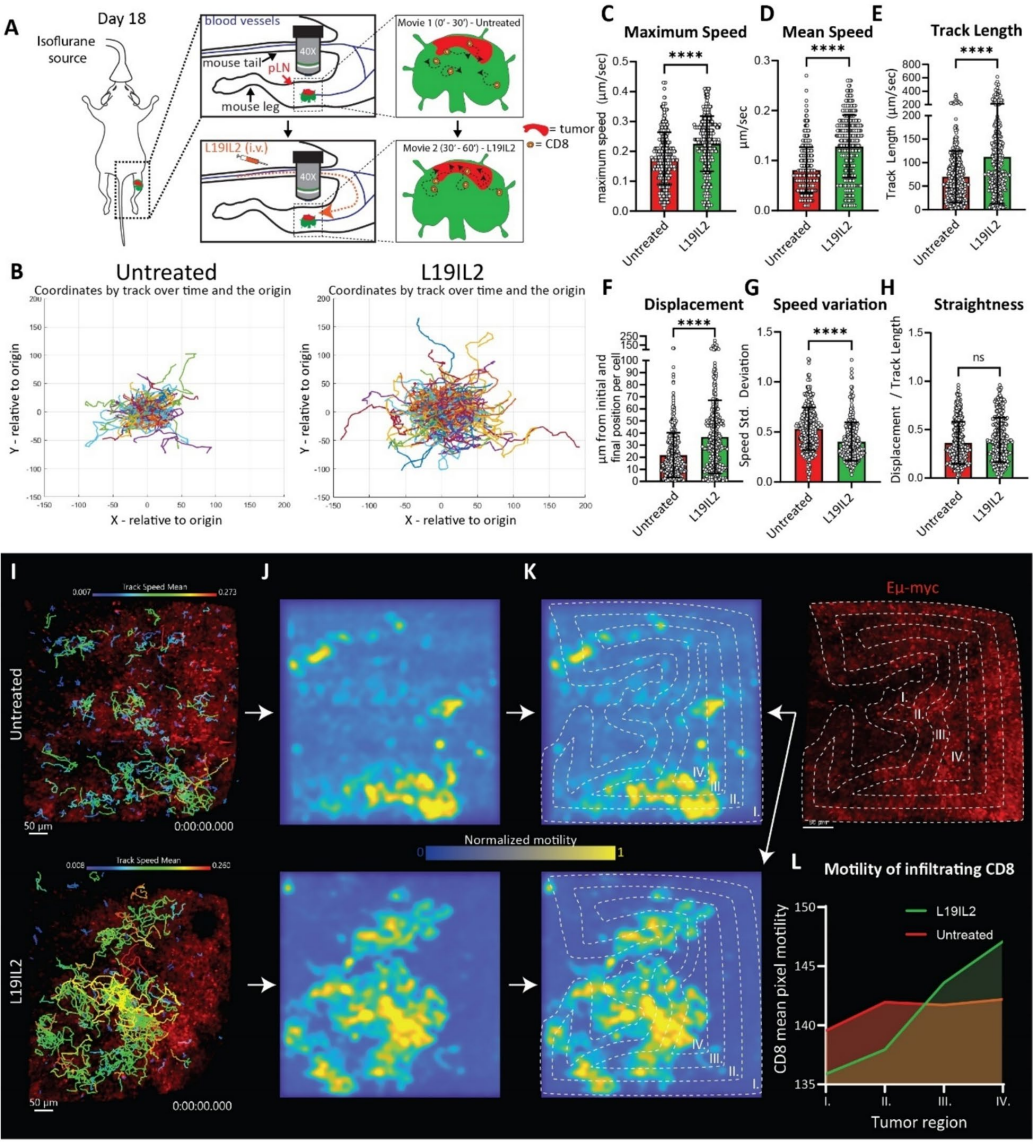
**A** Schematic representation of the 2-photon intravital microscopy (2P-IVM). **B** Plots of cell tracks with common origin showing longer tracks in L19IL2 treated CD8^+^ T cells in comparison to the same field of view before L19IL2 administration. Cell motility quantification using track maximum (**C**) speed, (**D**) mean speed, (**E**) length, (**F**) displacement, (**G**) speed variation, and (**H**) straightness. **I** CD8^+^ T cell tracks in untreated (up) and at 30 min post treatment (down) relative to lymphoma cells (red). **J** Pixel motility heatmap in untreated (up) and treated mice (down) showing hotspots with high motility (yellow). **K** right Equidistant regions of interest (ROIs) showing the different tumor regions from peripheral (I.) to deep areas (IV.), applied to pixel motility heatmap (left; up untreated and down L19IL2 treated). **L** Quantification of pixel velocity for each concentric ROI before (red) and after (green) L19IL2 administration. In (**C**-**H**) circles represent individual track values of all CD8.^+^ T cells from one representative mouse. In all graphs, the *p*-value is indicated as * < 0.05; ** < 0.01; *** < 0.001; **** < 0.0001

To evaluate the impact of L19IL2 on the dynamic behavior of CD8⁺ T cells, we applied 2-photon intravital microscopy (2P-IVM) to record lymphoma lesions in the pLN at 18 dpi (Fig. 6A, Supp. Mov. 1) and extract the CD8⁺ T cells tracks (Fig. 6B) before and after L19IL2 injection. This analysis highlighted that L19IL2 induced an increase in CD8⁺ maximum speed (Fig. 6C), mean speed (Fig. 6D), track length, which is the total distance covered by a cell in the recording (Fig. 6E), and track displacement, which indicates the distance between the initial and final position of a cell in a timelapse (Fig. 6F). Differently, speed variation, which indicates the heterogeneity in the speed of CD8⁺ T cells, decreased after therapy (Fig. 6G). Moreover, CD8⁺ T straightness, which indicates how directional are the overall cell movements, was not impacted by L19IL2 (Fig. 6H). To verify if L19IL2 induces a chemokine gradient influencing these motility parameters, we measured by flow cytometry the concentration of 13 chemokines in the LN supernatant (Supp. Figure 6 A). This analysis highlighted no differences between L19IL2 and untreated animals with respect to T cells related chemokines, including CXCL9 and CXCL10. Differently, CXCL5, which is not known to have a major role in T cell motility, was upregulated by L19IL2.

To further characterize the spatial motility of CD8⁺ T cells, we first applied a motility hot-spot detection tool [38]. This analysis measures CD8⁺ T cells motility (F ig. 6I) by quantifying the velocity in the CD8⁺ T cells channel for each pixel in time (Supp. Figure 6B) and representing it in a 2D graph on a colorimetric scale (Fig. 6J). In this representation, high colorimetric values indicate high CD8⁺ T cells motility and vice versa. Next, to assess the regional distribution of CD8^+^ T cells motility from the tumor border, we created multiple inner equidistant (40 μm) regions of interest (ROI) starting from an external predefined one, drawn around the tumor (Fig. 6K). Finally, we measured the average CD8⁺ T cells motility at a pixel level for each ROI before and after L19IL2 (Fig. 6L). This approach highlighted that CD8⁺ T cells moved equally in all the areas in untreated conditions, but their motility polarized from the tumor edge (periphery or margin) into the core of the tumor lesion following L19IL2, suggesting a tumor-oriented pattern of infiltration.

### L19IL2 reprograms CD8⁺ T cells spatial behavior towards tumor-directed patterns

**Fig. 7:**
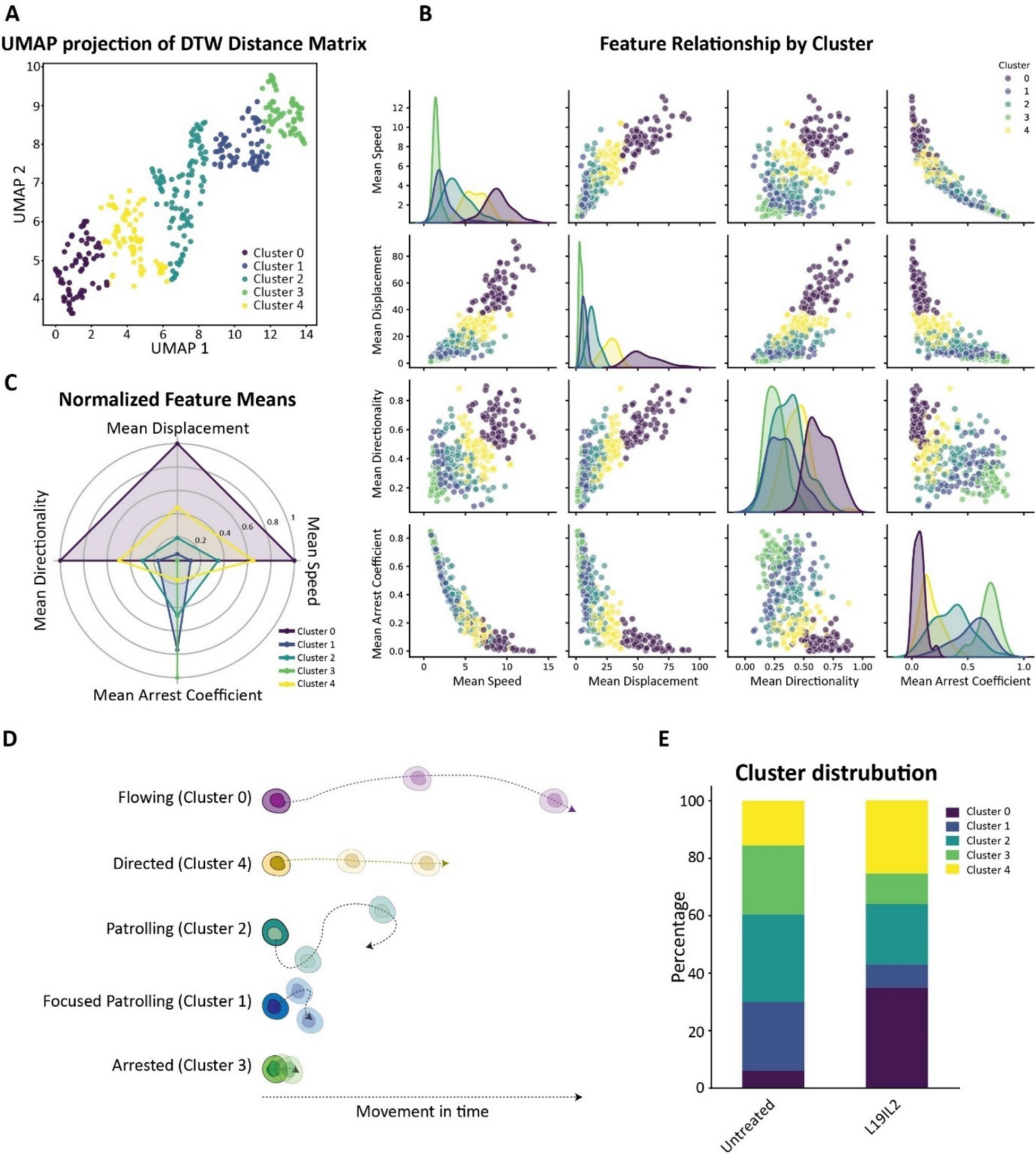
**A** UMAP graph of CD8^+^ T cells tracks from the same mouse before (untreated) and after L19IL2 treatment, pooled. The UMAP graph shows the clustering of CD8^+^ T cell tracks into five distinct groups, based on the dynamic time warping (DTW) distance matrix. **B **Pair plot visualizing the relationships between mean speed, mean displacement, mean directionality, and mean arrest coefficient across the five clusters of CD8^+^ T cells tracks. **C** Radar chart representing the average value of each parameter in the track clusters, representing differences between clusters. **D** Schematic representation of the motile patterns corresponding to the parameters measured in each cluster and the corresponding biological interpretation. **E** Percentage of CD8^+^ T cell tracks assigned to each cluster in the L19IL2 treated versus untreated conditions

To better understand CD8⁺ T cells post therapy behavior, we followed a computational approach to analyze the actions of leukocytes in the 2P-IVM recordings [38]. This method employs different cellular motility parameters to deduct the behavior or action that a cell is performing in a given time. Additionally, we implemented this method with a dynamic time warping algorithm, to cluster CD8⁺ T cell tracks regardless of the track duration [36]. This approach generated a cross-distance matrix by analyzing speed, directionality, displacement, and arrest coefficient of each track (Supp. Figure 7 A). Tracks were later clustered in a UMAP plot (Fig. 7A). Thus, each cluster shows distinct average values for each of the parameters evaluated (Fig. 7B). We then associated the multiparametric profile of each cluster (Fig. 7C) with the likely corresponding action (Fig. 7D). This analysis was based on classification criteria previously used [38]. In particular, we identified cluster 0 as flowing cells, with high speed, directionality, and displacement, but the lowest arrest coefficient, opposite to Cluster 4, which is defined as directed cells presenting intermediate to high values of speed, directionality, and displacement, and a low arrest coefficient. Cluster 2, instead, was characterized by intermediate values of all the parameters investigated, resembling the previously defined patrolling behavior [38]. Cluster 1 showed higher arrest coefficient and low speed, directionality, and displacement, thus being defined as focused patrolling. Finally, cluster 3 was populated by cells with extremely high arrest coefficient and no motility, compatible with an arrested phenotype. By comparing the percentage of tracks belonging to each cluster in L19IL2 versus untreated conditions (Supp. Figure 7B), we observed that L19IL2 induced an increase in the number of flowing (cluster 0) and directed (cluster 4) cells (Fig. 7E), supporting the hypothesis of a dynamic tumor directed activation of CD8⁺ T cells induced by L19IL2.

## Discussion

In this study, we established an in vivo model of DLBCL harboring a *myc* mutation, a genetic alteration frequently associated with aggressive disease phenotypes in both experimental and clinical settings [39]. The dissemination pattern observed in our model was characterized by preferential early colonization of the BM, preceding involvement of LN and spleen. While this pattern is uncommon in human DLBCL [40], it recapitulates the tropism observed in murine *myc*-driven lymphomas [41, 42]. Moreover, *myc* has been observed to be an inducer of angiogenesis and lymphangiogenesis [43], supporting the validity of this model for studying the mechanisms of action of neovessels-based therapies.

The potent antitumoral effect of L19IL2 observed in our model in multiple organs corroborates the application of this targeted approach for late-stage diseases. Though the majority of our experiments characterized the effect of L19IL2 on the spleen and LN microenvironment. Notably, LN tumor lesions are prevalent across the majority of cancers, including solid tumors, where LN serve as the initial site of metastasis [44, 45]. Thus, it is reasonable to speculate that the mechanisms described herein may extend to other tumors characterized by EDB^+^ neovessel, advocating for the use of L19IL2 in other cancers, especially during the metastatic phases. Importantly, antitumoral properties of L19IL2 were demonstrated in various tumors, including teratocarcinoma [16], lung cancer [16], colon adenocarcinoma [16], colorectal cancer [46], melanoma [47], and pancreatic ductal adenocarcinoma [48].

The enhanced tumor control occurring in the BM, which is devoid of mature, differentiated cytotoxic cells, poses questions about the mechanisms behind it. One possible explanation is that L19IL2 induces the recruitment of activated CD8^+^ T cells from the LN and spleen to other lymphoma-invaded organs. This hypothesis is supported by previous observations showing that local subcutaneous administration of L19IL2 induces regression of distant uninjected lesions [21]. This study suggests a systemic mobilization of cytotoxic cells from the site of L19IL2 accumulation to other anatomical regions, a mechanism that might be shared with our model.

The use of human IL2 in murine models represents a potential confounding factor. Nevertheless, due to its similarity to murine IL2, human IL2 typically cross-reacts with murine IL2 receptors. For this reason, human IL2 is widely employed in mouse studies, including investigations of clinically relevant immunocytokines [49]. Importantly, at the administration protocol that we used here, L19IL2 was previously shown to be non-immunogenic in vivo [47], enabling extensive preclinical validation [18, 50] and supporting its advancement to phase III clinical trials.

Our data identify CD8^+^ T cells as the primary mediators of the antitumoral effects of L19IL2. This is partially in contrast with a previous study demonstrating that F8IL2, an immunocytokine delivering IL2 through an EDA-targeting approach, mediates colon cancer regression through a combined action of CD8^+^ T cells and NK cells [37]. Discrepancies in the experimental design, including the use of different antibody delivery systems, combination with checkpoint inhibitors, and validation in distinct cancer models, may account for these contrasting results. Nevertheless, our data do not exclude a role for NK cells. Indeed, NK cell depletion accelerated lymphoma progression in our model, supporting the well-established role of these cells in lymphoma control [51]. It is plausible that NK contribution to L19IL2 efficacy is modulated by their basal activation status, with NK cells not requiring additional IL2 to trigger tumor killing. In contrast, CD8^+^ T cells, often dysfunctional in DLBCL, as demonstrated by the limited effect of their depletion on lymphoma growth, may be more susceptible to activation therapies in this context [52]. Additionally, previous evidence of limited LN homing capacity by NK cells might explain their poor response to therapy in this organ [53].

Although IL2 is known to activate CD8^+^ T cells, the lack of T_reg_ activation remains unexplained, considering their expression of high affinity IL2 receptor. Several factors may contribute to this selective CD8^+^ T cell activation. First, T_reg_ spatial distribution, particularly their localization near peritumoral lymphatic vessels, is critical for their interaction with regulatory DCs and suppression of CD8^+^ T cell-mediated antitumor immunity [54]. Intravenous administration of L19IL2 may preferentially target blood vessels over lymphatic vessels, potentially limiting its impact on T_reg_. Moreover, T_reg_ are known to promote neoangiogenesis [55], which could enhance the presence of L19IL2 targets and its subsequent effect on CD8^+^ T cells. Additionally, a recent study has described two distinct priming phases of CD8^+^ T cell immunity, with the second phase being crucial for CD8^+^ T cells activation and limited by T_reg_-mediated IL2 deprivation [56]. Administering exogenous IL2 during this phase could provide CD8^+^ T cells with sufficient IL2 for activation and circumvent T_reg_-mediated suppression. Moreover, the second phase of CD8⁺ T cell activation is facilitated by CXCR3 expression, which enhances CD8⁺ cell access to IL2–rich regions, consistent with the upregulation of CXCR3 in CD8⁺ T cells following L19IL2 treatment. This mechanism may lead to a positive feedback loop, wherein L19IL2 signaling induces IL2 receptor alpha and CXCR3 expression on CD8^+^ T cells, enhancing their sensitivity to IL2. Notably, additional factors have been linked to improved IL2 responsiveness. These include prolonged dwelling time, typically greater in CD8⁺ than in T_reg_ cells [56], and TCR engagement, which has been shown to enhance CD8⁺ effector fitness in response to IL2 [57].

The specificity of L19IL2 for CD8^+^ T cells is further supported by its greater activation of CD8^+^ T cells compared to unconjugated IL2. Previous studies have indicated that L19IL2 has a reduced effect on T_reg_ activation [17]. This effect was explained by different binding avidity towards the dimeric form L19IL2, which we used here, in contrast to monomeric L19IL2 [50]. This evidence aligns with the broader concept that protein structure influences receptor interactions and functional outcomes, a principle leveraged to design therapeutic molecules with minimal off-target effects, particularly in the case of IL2 [58]. For example, different complexes, such as IL2 bound to anti-IL2 antibodies, showed increased activity on T_reg_ [59–62]. Conversely, L19IL2 does not activate T_reg_. Thus, it is possible to hypothesize a polarized activity of dimeric L19IL2 towards CD8⁺ T cells over T_reg_, which may justify both the lack of T_reg_ activation observed in our in vivo model and the superior activation of CD8⁺ T cells relative to unconjugated IL2 in vitro. These findings strongly support the clinical value of dimeric L19IL2 as a strategy to selectively enhance cytotoxic responses while minimizing regulatory constraints.

The apparent differences between CD8^+^ T cell phenotypes observed in vitro and in vivo primarily reflect distinct temporal stages of differentiation. In vitro analyses recapitulate early activation, characterized by the expansion of stem-like and memory-like cells, whereas in vivo experiments represent a later phase following sustained antigen exposure, with a predominance of terminally differentiated effector cells that are critical for tumor killing. In vivo PD1 expression was low and, in the absence of additional markers such as TIM-3, LAG-3, or TIGIT, is insufficient to define exhaustion [63]. By contrast, early co-expression of PD1 and TCF1 identifies stem-like or memory-like subsets essential for durable immune responses [64, 65]. Similarly, TOX marks stem-like CD8 T cells when co-expressed with TCF1 [66], while indicating exhaustion only in TCF1⁻ cells [67]. Together, our in vitro and in vivo data demonstrate that L19–IL2 promotes both early memory-like and later effector CD8^+^ T cell programs, resulting in a coordinated and sustained antitumor immune response.

In this study, we have not clarified if the increased CD8^+^ T cells priming is due to a combined effect of L19IL2 on DCs and CD8^+^ T cells or only on one of the two populations. The overexpression of CD80 by DCs might suggest that the upregulation of this costimulatory receptor is induced by L19IL2. Supporting this hypothesis, previous evidence has shown that IL2 stimulates DCs, eventually promoting antitumor immunity [68]. Alternatively, the lowering of the TCR activation threshold of the CD8^+^ T cells could also explain their increased priming. This effect was previously observed to be induced by IL2 in CD8^+^ but not CD4^+^ T cells [69].

The observation of increased CD8^+^ T cell priming provides a rationale for combining L19IL2 with immunotherapies that promote TCR activation, such as checkpoint inhibitors or cancer vaccines. A question of future interest is whether L19IL2 may also enhance therapeutic efficacy in relapses or tumors resistant to classical therapies. In these conditions, CAR T cells are often used as second-line therapy for lymphoma [70]. One of the limitations of CAR T is that certain tumors can limit their cytotoxic function by inducing an immune suppressive microenvironment [71]. Interestingly, some authors proposed targeted IL2 as a strategy to circumvent this immunosuppressive mechanism and improve CAR T efficiency [72], advocating for further investigations on the effect of L19IL2 in relapsing tumors, alone or in combination with CAR T.

By employing advanced intravital microscopy we elucidated the dynamic activation of CD8^+^ T cells within tumors before and after L19IL2 administration. This approach provided internal controls, reducing inter-animal variability. The rapid delivery of L19IL2 to the tumor [18] together with the quick action of IL2 on CD8^+^ T cells [73] facilitated real-time imaging of L19IL2 effects on immune cells. Moreover, we applied advanced computational methodologies, including track clustering, correction for track duration–related bias, and multiparametric analysis of CD8⁺ T cell motility, to investigate the spatial behavior of T cells in tumor tissues. A similar approach was previously used to investigate cytotoxic cell behavior in vitro [36]. However, no reports have studied the actions of tumor infiltrating CD8^+^ T cells in vivo. This spatial behavior analysis revealed that L19IL2 activated CD8^+^ T cells, which adopted a more dynamic and tumor-oriented motility. This finding aligns with previous reports indicating that IL2 activated CD8^+^ T cells rapidly increase motility [34] to enhance target cell encounters [74]. Despite cytotoxic killing is a process that involves intricate interactions [75], prior studies have shown that effective killing often results from multiple short, sublethal contacts rather than prolonged engagements [76]. These observations are consistent with the increased directional behavior observed following L19IL2 treatment, which further demonstrates that tumor killing is associated with increased motility specifically within the target lesions. Moreover, the increase in flowing behavior suggests an enhanced recruitment of CD8⁺ T cells, which is in line with the elevated numbers observed by flow cytometry.

Importantly, we demonstrated that L19IL2 induced changes in CD8^+^ T cell motility and CXCR3 expression in the absence of major upregulation of T cells chemokines. CXCR3 upregulation may enhance CD8^+^ T cell sensitivity to existing chemokine gradients [25], facilitating their migration toward tumor sites. Additionally, T cells can modify their dynamic behavior through chemokine-independent, cell-intrinsic factors induced by activation [74], which may also play a role in our model.

## Conclusion

Collectively, our data demonstrate that L19IL2 serves as an effective platform for delivering IL2 specifically to tumor-infiltrating CD8^+^ T cells, enhancing their cytotoxic capacity and addressing several challenges associated with IL2 therapy, including systemic toxicity and lack of specificity for CD8^+^ T cells.

These findings provide a strong foundation for exploring synergistic combinations of L19IL2 with other immunotherapies, including checkpoint inhibitors, CAR T cells, and cancer vaccines, in the treatment of lymphoma and other tumors. Furthermore, we explored the potential of targeted IL2 to expand private CD8^+^ T cell clones and control the spatial behavior of CD8^+^ T cells within tumors.

## Supplementary Information

Supplementary Material 1: Supplementary Figure 1. (A) Histogram showing mCherry expression in Eμ-myc wild type (wt, blue) and Eμ-myc mCherry transduced (red) cells. (B) Scatter plots showing the flow cytometry gating strategy used to quantify lymphoma cells, defined as live dead^-^CD45.2^+^B220^+^ mCherry^+^. (C) Flow cytometric quantification of lymphoma cells in the bone marrow (BM) in untreated and L19IL2 treated animals. Flow cytometric quantification of lymphoma cells in (D) the spleen and (E) BM in untreated versus L19IL2 or L19mTNF treated animals. In (C), (D), and (E), circles represent individual values (animal replicates), while the red line indicates the average value per group. In (C) the dashed red line shows the background value in non-tumor injected animals. In all graphs, the p-value is indicated as * <0.05; ** <0.01; ***<0.001; **** < 0.0001. Supplementary Figure 2. (A) Flow cytometric quantification of CD8^+^ T cells in the pLN and spleen of CD8 depleted mice. (B) Flow cytometric quantification of NK cells in the pLN and spleen of NK depleted mice. (C) Effect of L19IL2 on the number of lymphoma cells in the pLN, measured by flow cytometry, in CD8 T cells depleted mice in comparison to controls (PBS). Data indicate results from three independent experiments, pooled. Each point shows the average value per group per experimental repetition. Results were normalized to the average value of PBS controls in each experiment. (D) Flow cytometric quantification of lymphoma cells in the spleen of NK depleted mice untreated compared to L19IL2 treatment. (E) Gating strategy to isolate NK, NK T, CD8^+^ T cells, CD4^+^ T cells, and T_reg_. Flow cytometric quantification of (F) CD11b^-^ and CD11b^+^ dendritic cells (DCs), (G) B cells, (H) Macrophages, and (I) Neutrophils in the LN of untreated and L19IL2 treated animals. (J) Flow cytometric histogram showing gating strategy to isolate Ki67^+^ cells. In all graphs circles represent individual values (animal replicates), while red lines or bars indicate the average value per group. In all graphs, the p-value is indicated as * <0.05; ** <0.01; ***<0.001; **** < 0.0001. Supplementary Figure 3. (A) Schematic representation of the lymphoma model used for T cells UMAP clustering, in which T cells parameters were acquired by using flow cytometry of the LN of lymphoma-bearing mice. (B) UMAP clustering of total CD3^+^ T cells, showing the expression of the subclusters identity markers across subsets in a colorimetric scale, including NK1.1, CD62L, CXCR3, CD44, and CD11b. UMAP representation of the T cells from (C) L19IL2 treated (blue) or (D) untreated (orange) mice. Circles indicate individual cells. (E) Schematic representation of the in vitro immunophenotyping experiment. (F) Percentage of CD127^+^CD8^+^ T cells in treated with L19IL2 or IL2 conditions. Circles indicate technical replicates, while lines show average and standard deviation per group. In all graphs, the p-value is indicated as * <0.05; ** <0.01; ***<0.001; **** < 0.0001. Supplementary Figure 4. (A) Volcano plot representing the differential gene expression in CD8^+^ T cells isolated from L19IL2 treated mice in comparison to untreated. The red dashed box contains all the significantly downregulated genes in L19IL2 (red dots, magnified on the right). Killing capacity of (B) LN and (C) spleen isolated CD8^+^ T cells, measured as the percentage of surviving lymphoma cells, quantified by flow cytometry, following 24 h (left) and 48 h (right) co-incubation, at different effector.target ratio. (D) Graphical representation of the construct used to transduce primary CD8^+^ T cells and generate anti-CD19 CAR T. In all graphs, the p-value is indicated as * <0.05; ** <0.01; ***<0.001; ****< 0.0001. Supplementary Figure 5. (A) Schematic representation of the experimental pipeline to isolate DNA from CD8^+^ T cells for TCR sequencing. (B) Nucleic acid sequences of the MPC from L19IL2 treated (1-4, green) and untreated (5-8, red) mice. (C) Mean RNA expression of the Cd28 gene in CD8^+^ T cells isolated from Untreated (red) or L19IL2 treated (green) mice. Bars indicate mean value and lines indicate standard deviation (n = 4). (D) Percentage of CD80^+^ DCs in the LN of untreated versus L19IL2 treated mice. Bars indicate mean value and lines indicate standard deviation (n = 4). Supplementary Figure 6. (A) Quantification of inflammatory chemokines in the supernatant of untreated (top row) and L19IL2 treated (bottom row) lymphoma-invaded LNs (n = 5). (B) Explanatory drawing representing the process of pixel velocity quantification. The variation of intensity of the CD8^+^ T cell fluorescent signal is used to extract pixel velocity and, consequently, the motion magnitude, correlating with a motile behavior in that area. Supplementary Figure 7. (A) Heatmap showing the pairwise correlations of the parameters used for UMAP clustering. Numbers indicate the index of correlation and are associated with a colorimetric scale. (B) Quantification of the percentage of tracks assigned to each cluster in the L19IL2 treated versus untreated conditions. Numbers indicate the percentage of total tracks in that group and inversely correlate with the darkness of the box color.

## Data Availability

All data are available from the corresponding author upon a reasonable request.
