# Supplementary material for: Tumor-targeted IL2 promotes specific CD8+ T cells private clonal expansion enhancing lymphoma control
Source: J Exp Clin Cancer Res. 2026 Mar 3;45:91. doi: 10.1186/s13046-026-03678-7 (PMC13064391; doi:10.1186/s13046-026-03678-7)
Supplement: Supplementary file 1 — Supplementary Material 1: Supplementary Figure 1. (A) Histogram showing mCherry expression in Eμ-myc wild type (wt, blue) and Eμ-myc mCherry transduced (red) cells. (B) Scatter plots showing the flow cytometry gating strategy used to quantify lymphoma cells, defined as live dead-CD45.2+B220+ mCherry+. (C) Flow cytometric quantification of lymphoma cells in the bone marrow (BM) in untreated and L19IL2 treated animals. Flow cytometric quantification of lymphoma cells in (D) the spleen and (E) BM in untreated versus L19IL2 or L19mTNF treated animals. In (C), (D), and (E), circles represent individual values (animal replicates), while the red line indicates the average value per group. In (C) the dashed red line shows the background value in non-tumor injected animals. In all graphs, the p-value is indicated as * <0.05; ** <0.01; ***<0.001; **** < 0.0001. Supplementary Figure 2. (A) Flow cytometric quantification of CD8+ T cells in the pLN and spleen of CD8 depleted mice. (B) Flow cytometric quantification of NK cells in the pLN and spleen of NK depleted mice. (C) Effect of L19IL2 on the number of lymphoma cells in the pLN, measured by flow cytometry, in CD8 T cells depleted mice in comparison to controls (PBS). Data indicate results from three independent experiments, pooled. Each point shows the average value per group per experimental repetition. Results were normalized to the average value of PBS controls in each experiment. (D) Flow cytometric quantification of lymphoma cells in the spleen of NK depleted mice untreated compared to L19IL2 treatment. (E) Gating strategy to isolate NK, NK T, CD8+ T cells, CD4+ T cells, and Treg. Flow cytometric quantification of (F) CD11b- and CD11b+ dendritic cells (DCs), (G) B cells, (H) Macrophages, and (I) Neutrophils in the LN of untreated and L19IL2 treated animals. (J) Flow cytometric histogram showing gating strategy to isolate Ki67+ cells. In all graphs circles represent individual values (animal replicates), while red lines [file 13046_2026_3678_MOESM1_ESM.docx]

**SUPPLEMENTARY FILES**


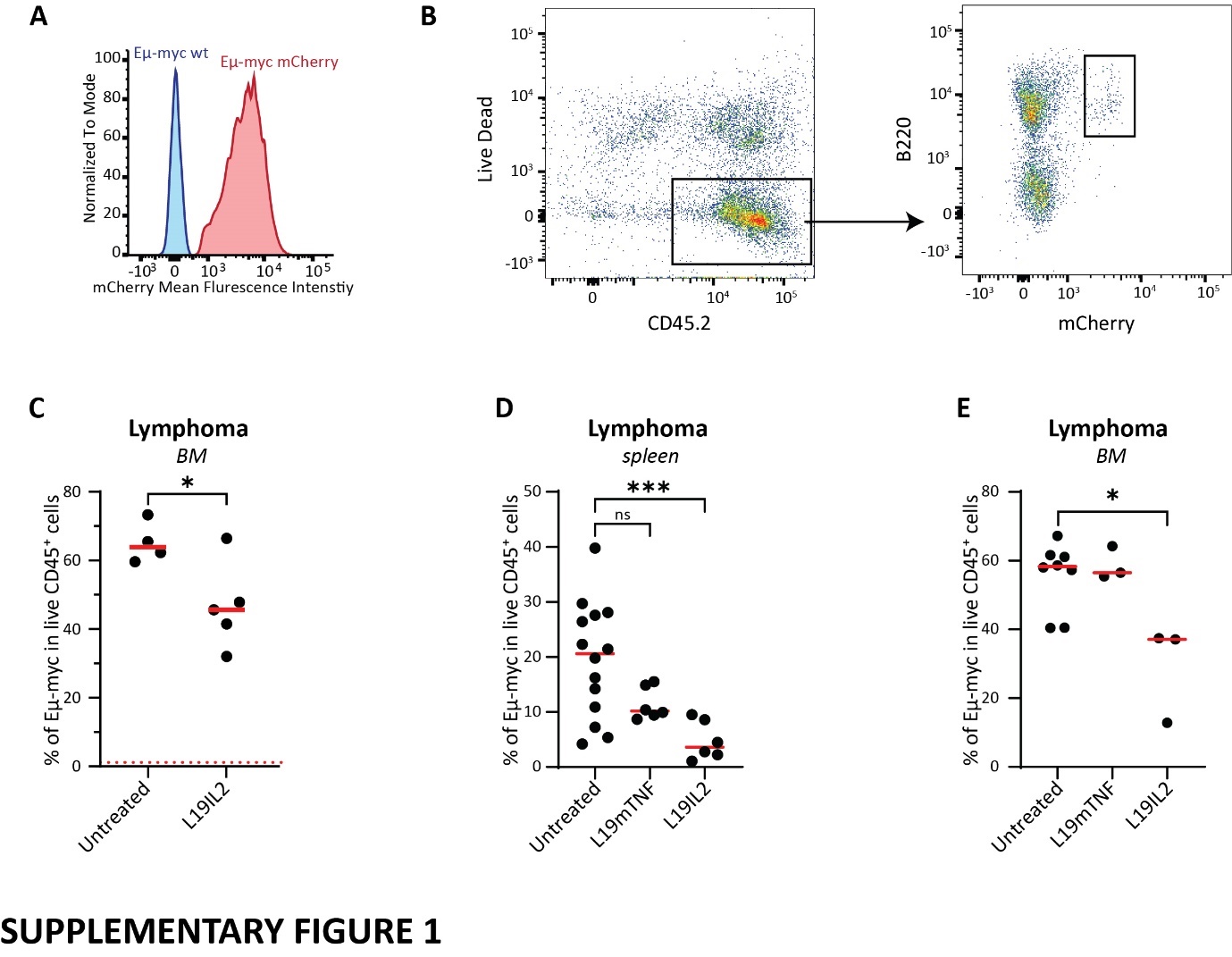
***Supplementary Figure 1.*** *(****A****) Histogram showing mCherry expression in Eμ-myc wild type (wt, blue) and Eμ-myc mCherry transduced (red) cells. (****B****) Scatter plots showing the flow cytometry gating strategy used to quantify lymphoma cells, defined as live dead^-^ CD45.2^+^ B220^+^ mCherry^+^. (****C****) Flow cytometric quantification of lymphoma cells in the bone marrow (BM) in untreated and L19IL2 treated animals. Flow cytometric quantification of lymphoma cells in (****D****) the spleen and (****E****) BM in untreated versus L19IL2 or L19mTNF treated animals. In (****C****), (****D****), and (****E****), circles represent individual values (animal replicates), while the red line indicates the average value per group. In (****C****) the dashed red line shows the background value in non-tumor injected animals. In all graphs, the p-value is indicated as * <0.05; ** <0.01; ***<0.001; **** < 0.0001.*


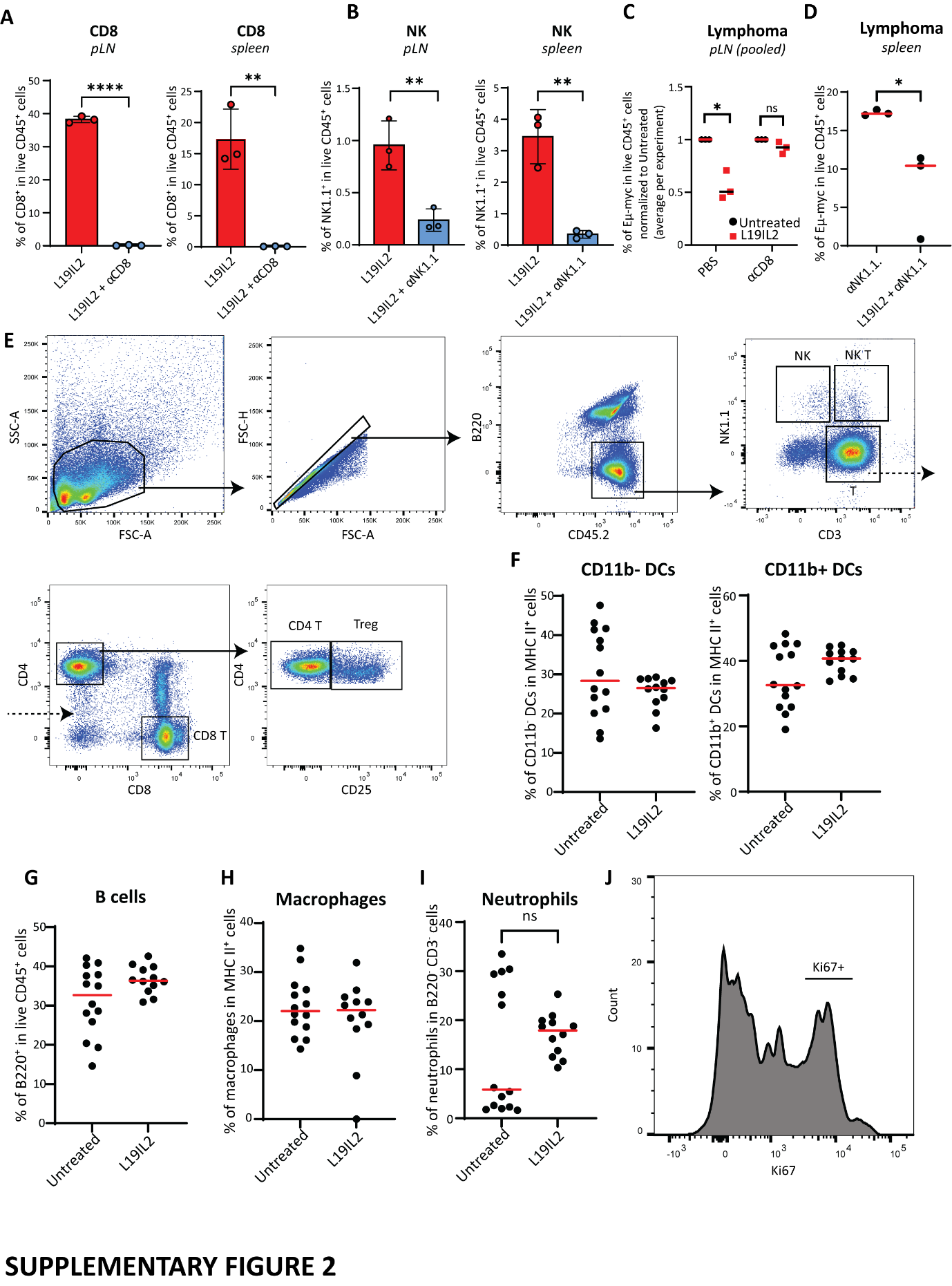


***Supplementary Figure 2.*** *(****A****) Flow cytometric quantification of CD8^+^ T cells in the pLN and spleen of CD8 depleted mice. (****B****) Flow cytometric quantification of NK cells in the pLN and spleen of NK depleted mice. (****C****) Effect of L19IL2 on the number of lymphoma cells in the pLN, measured by flow cytometry, in CD8 T cells depleted mice in comparison to controls (PBS). Data indicate results from three independent experiments, pooled. Each point shows the average value per group per experimental repetition. Results were normalized to the average value of PBS controls in each experiment. (****D****) Flow cytometric quantification of lymphoma cells in the spleen of NK depleted mice untreated compared to L19IL2 treatment. (****E****) Gating strategy to isolate NK, NK T, CD8^+^ T cells, CD4^+^ T cells, and T_reg_. Flow cytometric quantification of (****F****) CD11b^-^ and CD11b^+^ dendritic cells (DCs), (****G****) B cells, (****H****) Macrophages, and (****I****) Neutrophils in the LN of untreated and L19IL2 treated animals. (****J****) Flow cytometric histogram showing gating strategy to isolate Ki67^+^ cells. In all graphs circles represent individual values (animal replicates), while red lines or bars indicate the average value per group. In all graphs, the p-value is indicated as * <0.05; ** <0.01; ***<0.001; **** < 0.0001.*


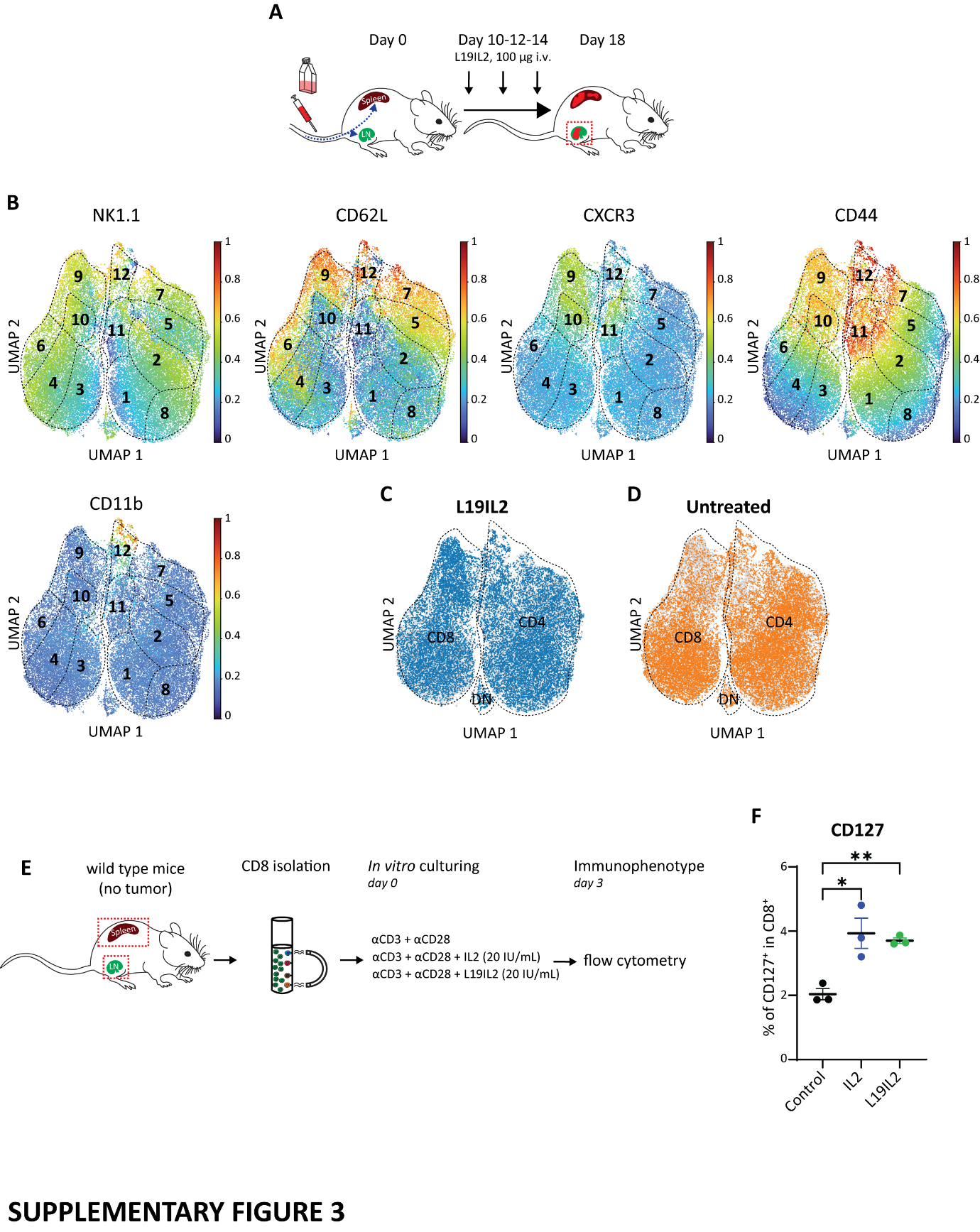
***Supplementary Figure 3.*** *(****A****) Schematic representation of the lymphoma model used for T cells UMAP clustering, in which T cells parameters were acquired by using flow cytometry of the LN of lymphoma-bearing mice. (****B****) UMAP clustering of total CD3^+^ T cells, showing the expression of the subclusters identity markers across subsets in a colorimetric scale, including NK1.1, CD62L, CXCR3, CD44, and CD11b. UMAP representation of the T cells from (****C****) L19IL2 treated (blue) or (****D****) untreated (orange) mice. Circles indicate individual cells. (****E****) Schematic representation of the* in vitro *immunophenotyping experiment. (****F****) Percentage of CD127^+^ CD8^+^ T cells in treated with L19IL2 or IL2 conditions. Circles indicate technical replicates, while lines show average and standard deviation per group. In all graphs, the p-value is indicated as * <0.05; ** <0.01; ***<0.001; **** < 0.0001.*


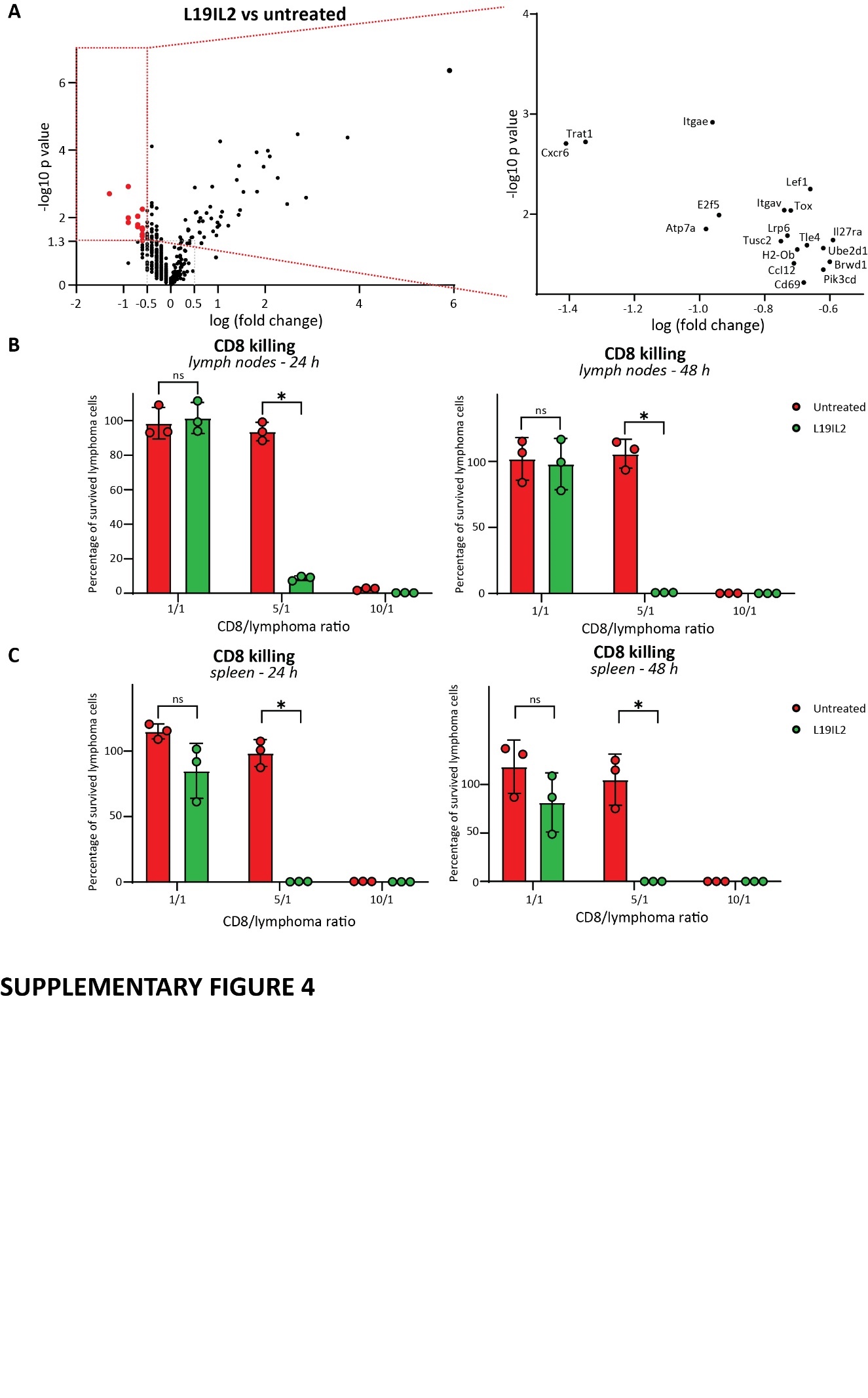


***Supplementary Figure 4.*** *(****A****) Volcano plot representing the differential gene expression in CD8^+^ T cells isolated from L19IL2 treated mice in comparison to untreated. The red dashed box contains all the significantly downregulated genes in L19IL2 (red dots, magnified on the right). Killing capacity of (****B****) LN and (****C****) spleen isolated CD8^+^ T cells, measured as the percentage of surviving lymphoma cells, quantified by flow cytometry, following 24 h (left) and 48 h (right) co-incubation, at different effector.target ratio. (****D****) Graphical representation of the construct used to transduce primary CD8^+^ T cells and generate anti-CD19 CAR T. In all graphs, the p-value is indicated as * <0.05; ** <0.01; ***<0.001; **** < 0.0001.*


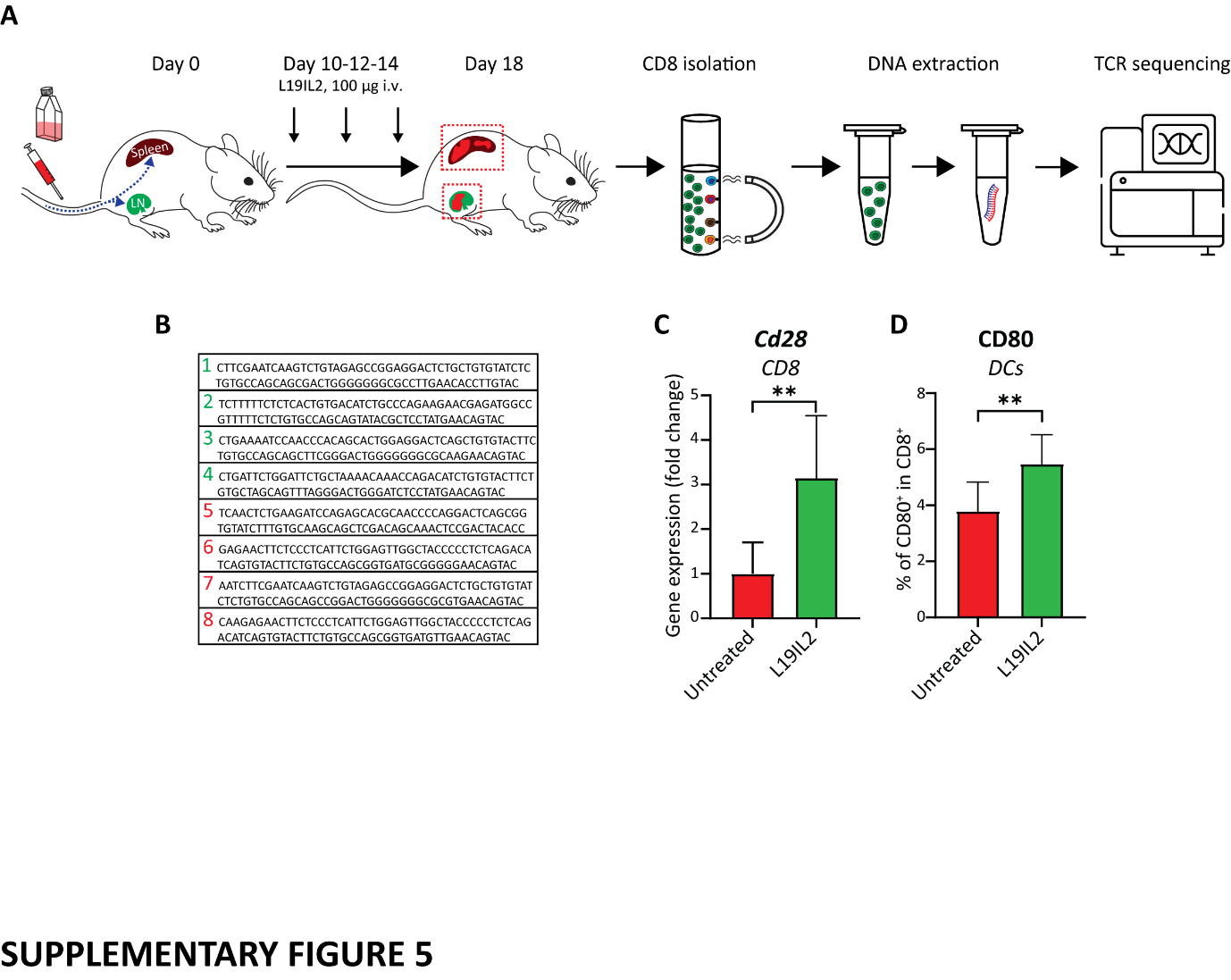


***Supplementary Figure 5.*** *(****A****) Schematic representation of the experimental pipeline to isolate DNA from CD8^+^ T cells for TCR sequencing. (****B****) Nucleic acid sequences of the MPC from L19IL2 treated (1-4, green) and untreated (5-8, red) mice. (****C****) Mean RNA expression of the* Cd28 *gene in CD8^+^ T cells isolated from Untreated (red) or L19IL2 treated (green) mice. Bars indicate mean value and lines indicate standard deviation (n = 4). (****D****) Percentage of CD80^+^ DCs in the LN of untreated versus L19IL2 treated mice. Bars indicate mean value and lines indicate standard deviation (n = 4).*


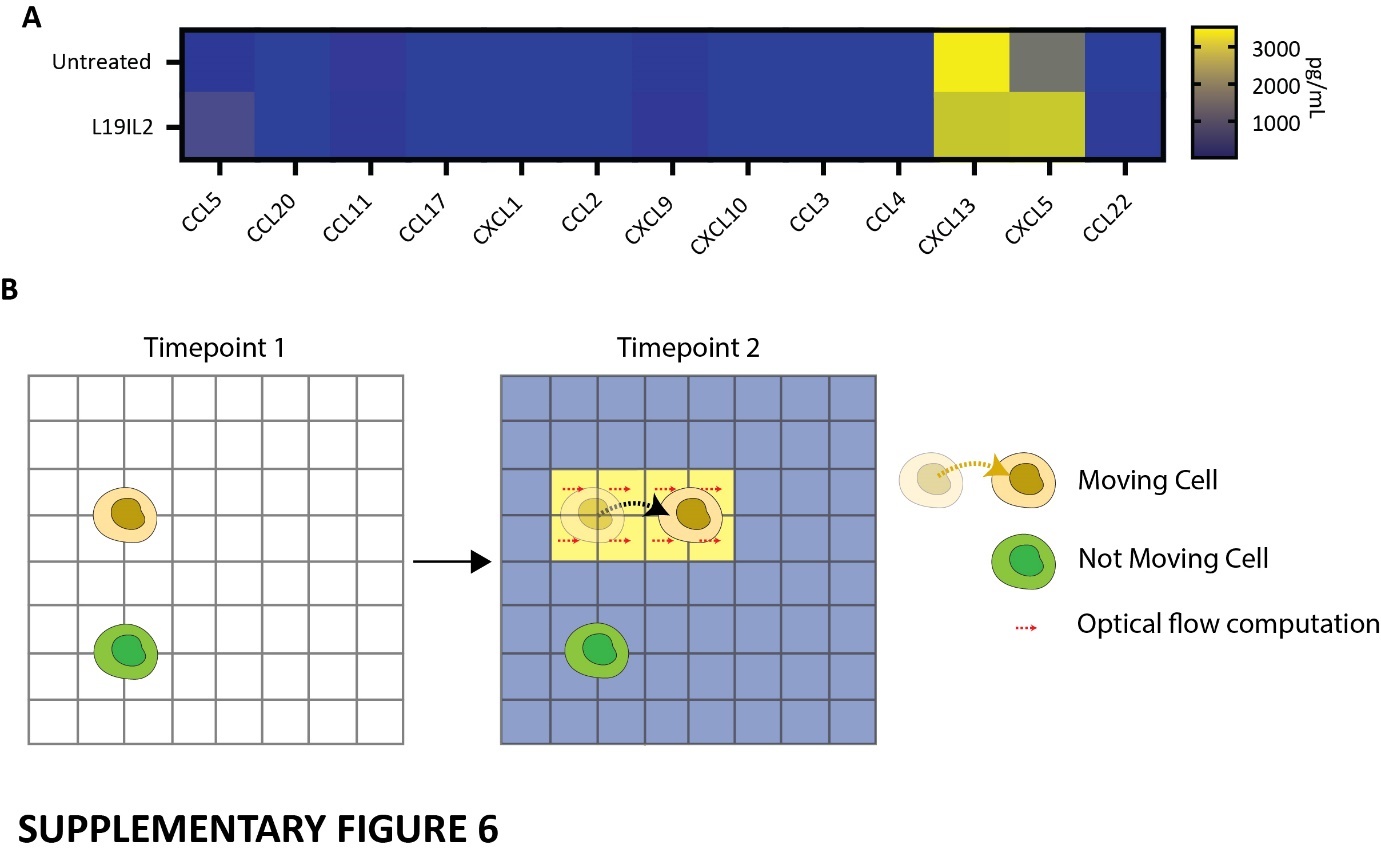


***Supplementary Figure 6.*** *(****A****) Quantification of inflammatory chemokines in the supernatant of untreated (top row) and L19IL2 treated (bottom row) lymphoma-invaded LNs (n = 5). (****B****) Explanatory drawing representing the process of pixel velocity quantification. The variation of intensity of the CD8^+^ T cell fluorescent signal is used to extract pixel velocity and, consequently, the motion magnitude, correlating with a motile behavior in that area.*


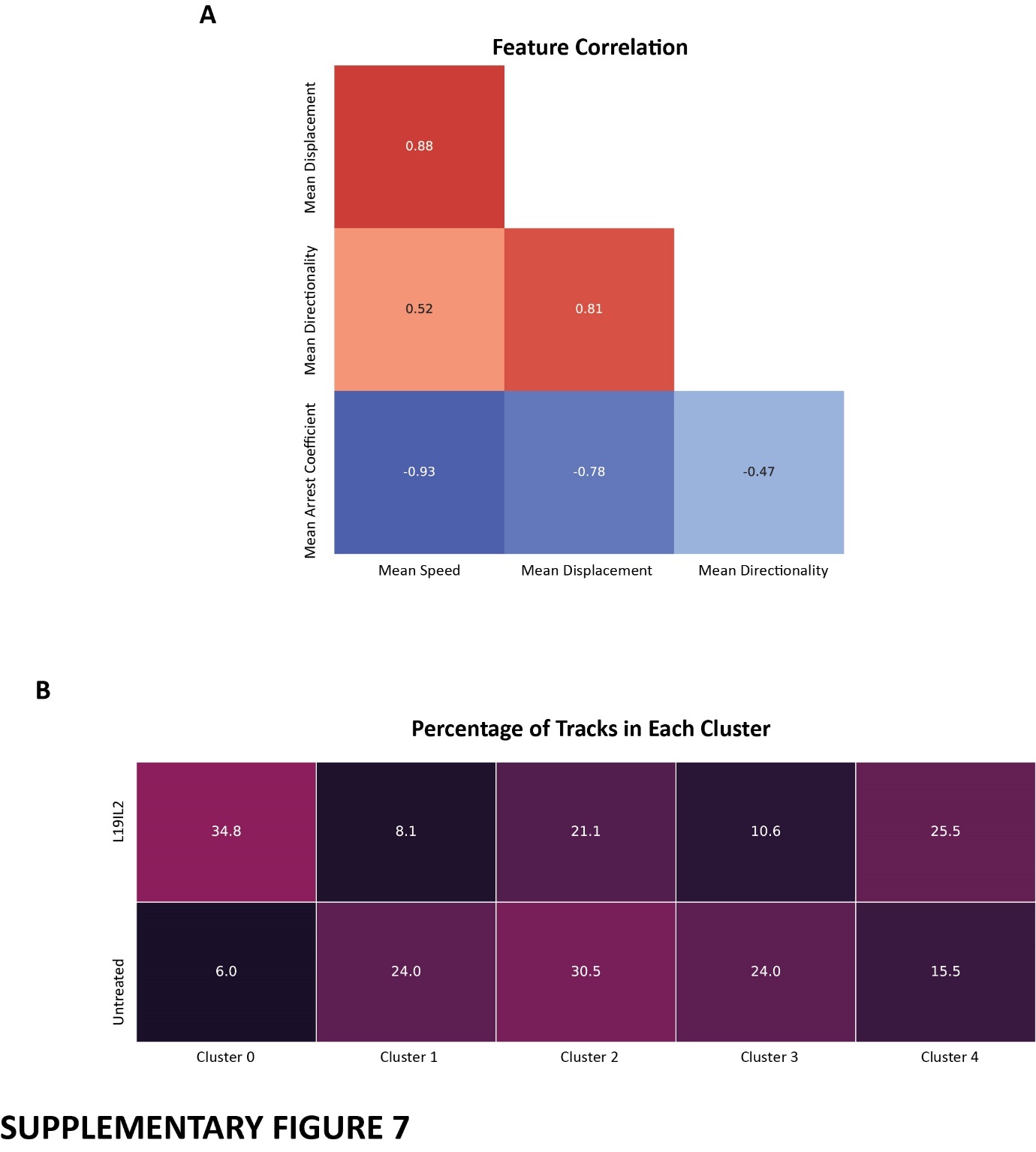


***Supplementary Figure 7.*** *(****A****) Heatmap showing the pairwise correlations of the parameters used for UMAP clustering. Numbers indicate the index of correlation and are associated with a colorimetric scale. (****B****) Quantification of the percentage of tracks assigned to each cluster in the L19IL2 treated versus untreated conditions. Numbers indicate the percentage of total tracks in that group and inversely correlate with the darkness of the box color.*

***Supplementary Movie 1.***

[**Supp. Movie 1.mp4**](https://usi365-my.sharepoint.com/:v:/g/personal/chahik_usi_ch/IQDbn_SdadnHR6ZkM0wMyx8tAebbR7xP6GfEIwP5rKHiStY?nav=eyJyZWZlcnJhbEluZm8iOnsicmVmZXJyYWxBcHAiOiJPbmVEcml2ZUZvckJ1c2luZXNzIiwicmVmZXJyYWxBcHBQbGF0Zm9ybSI6IldlYiIsInJlZmVycmFsTW9kZSI6InZpZXciLCJyZWZlcnJhbFZpZXciOiJNeUZpbGVzTGlua0NvcHkifX0&e=hihd6L)
